# Transcriptomic resources for the medicinal legume *Mucuna pruriens*: *de novo* transcriptome assembly, annotation, identification and validation of EST-SSR markers

**DOI:** 10.1186/s12864-017-3780-9

**Published:** 2017-05-25

**Authors:** N. Sathyanarayana, Ranjith Kumar Pittala, Pankaj Kumar Tripathi, Ratan Chopra, Heikham Russiachand Singh, Vikas Belamkar, Pardeep Kumar Bhardwaj, Jeff J. Doyle, Ashley N. Egan

**Affiliations:** 1 Department of Botany, Sikkim University, 6th Mile, Tadong-737102, Gangtok, Sikkim India; 2 United States Department of Agriculture, Agriculture Research Service, 3810 4th St., Lubbock, TX 79415 USA; 3 Department of Plant Science, McGill University, Raymond Building, 21111 Lakeshore Road, Ste. Anne de Bellevue, Quebec, H9X 3V9 Canada; 4 Department of Agronomy and Horticulture, University of Nebraska-Lincoln, Lincoln, NE 68583 USA; 5 Institute of Bioresources and Sustainable Development, ikkim Centre, Tadong-737102, Gangtok, Sikkim India; 6 Section of Plant Breeding and Genetics, School of Integrative Plant Science, Cornell University, 412 Mann Library, Ithaca, NY 14853 USA; 7 Department of Botany, Smithsonian Institution, National Museum of Natural History, US National Herbarium, 10th and Constitution Ave NW, Washington, DC 20013 USA

**Keywords:** Velvet bean, *Mucuna pruriens*, Transcriptomics, Differential gene expression, EST-SSRs, Population structure, Leguminosae, Fabaceae

## Abstract

**Background:**

The medicinal legume *Mucuna pruriens* (L.) DC. has attracted attention worldwide as a source of the anti-Parkinson’s drug L-Dopa. It is also a popular green manure cover crop that offers many agronomic benefits including high protein content, nitrogen fixation and soil nutrients. The plant currently lacks genomic resources and there is limited knowledge on gene expression, metabolic pathways, and genetics of secondary metabolite production. Here, we present transcriptomic resources for *M. pruriens*, including a *de novo* transcriptome assembly and annotation, as well as differential transcript expression analyses between root, leaf, and pod tissues. We also develop microsatellite markers and analyze genetic diversity and population structure within a set of Indian germplasm accessions.

**Results:**

One-hundred ninety-one million two hundred thirty-three thousand two hundred forty-two bp cleaned reads were assembled into 67,561 transcripts with mean length of 626 bp and N50 of 987 bp. Assembled sequences were annotated using BLASTX against public databases with over 80% of transcripts annotated. We identified 7,493 simple sequence repeat (SSR) motifs, including 787 polymorphic repeats between the parents of a mapping population. 134 SSRs from expressed sequenced tags (ESTs) were screened against 23 *M. pruriens* accessions from India, with 52 EST-SSRs retained after quality control. Population structure analysis using a Bayesian framework implemented in fastSTRUCTURE showed nearly similar groupings as with distance-based (neighbor-joining) and principal component analyses, with most of the accessions clustering per geographical origins. Pair-wise comparison of transcript expression in leaves, roots and pods identified 4,387 differentially expressed transcripts with the highest number occurring between roots and leaves. Differentially expressed transcripts were enriched with transcription factors and transcripts annotated as belonging to secondary metabolite pathways.

**Conclusions:**

The *M. pruriens* transcriptomic resources generated in this study provide foundational resources for gene discovery and development of molecular markers. Polymorphic SSRs identified can be used for genetic diversity, marker-trait analyses, and development of functional markers for crop improvement. The results of differential expression studies can be used to investigate genes involved in L-Dopa synthesis and other key metabolic pathways in *M. pruriens*.

**Electronic supplementary material:**

The online version of this article (doi:10.1186/s12864-017-3780-9) contains supplementary material, which is available to authorized users.

## Background

There are many minor food legumes whose potential is underexploited and untapped. Adzuki bean [*Vigna angularis* (Willd.) Ohwi & Ohashi], velvet or itching bean (*Mucuna* spp.), bambara groundnut (*Vigna subterranea* L.), faba bean (*Vicia faba* L.), horse gram [*Macrotyloma uniflorum* (Lam.) Verdc.], hyacinth bean *(Lablab purpureus* L.*)*, grass pea (*Lathyrus sativus* L.), moth bean [*Vigna aconitifolia* (Jacq.)], rice bean [*Vigna umbellata* (Thunb.) Ohwi and Ohashi] and winged bean [*Psophocarpus tetragonolobus* (L.) DC.] are prominent members of this group [1]. Many of them possess rich nutritional value and form an important source of protein, vitamins and minerals in low-income, food-deficit countries [2]. Being well adapted to marginal conditions, they may serve as storehouses of vital genes related to biotic and abiotic stress tolerance. Developing genomic resources and characterizing these important agronomic traits would help in identifying genes which could potentially be used in targeting other legumes to increase their tolerance.

*Mucuna* Adans. comprises 105 species [3] and is classified within the phaseoloid clade of Fabaceae, which also includes soybean, common bean, mung bean and other relatives [4]. *Mucuna pruriens* (L.) DC. (velvet bean) is reported to be native to China and East India [5] but has now attained a pantropical distribution with a major niche in the Indian sub-continent [6]. Like common bean and mung bean, velvet bean has a chromosome number of 2n = 2x = 22 [7], but with a much larger estimated genome size of between 1,281 to 1,361 Mbp/C (A.N. Egan & N. Sathyanarayana, unpublished data). The plant exhibits a climbing habit, hairy aerial parts and a long inflorescence of white or dark purple flowers. Pods are mostly green or brown in color with 4-6 seeds. In wild plants, the pods are thickly covered with soft or stiff orange bristles that cause intense allergic irritation to human skin in vars. *pruriens* and *hirsuta* whereas the cultivated var. *utilis* has non-irritant hairs. Botanically, it is represented by two varieties, var. *utilis* (cultivated) and var. *pruriens* (wild) - while the presence of a third group, var. *hirsuta* (wild), is also reported [8]. Owing to a wide-ranging geographical and climatic distribution, the species exhibits rich phenotypic diversity, especially in the Indian sub-continent.

The proximate nutritional composition, total protein content and in vitro protein digestibility of *M. pruriens* seeds are similar to other edible legumes [9]. Consequently, it is used as a minor food crop by native peoples of India and Africa while its use continues as livestock feed – a common use in the early 1900s in the USA [5]. It is known to produce seed yield of 2,000 kg/hectare [5], perform well under dry farming and low soil fertility conditions [10], exhibits allelopathic properties [11], and is effective in lowering pathogenic nematode populations [12]. Positive impacts of *M. pruriens* as a green manure cover crop are well documented in earlier studies [13]*.* The fast-growing habit of *M. pruriens* allows ground-cover within 60-90 days, producing large biomass vis-à-vis other cover crops [12]. This, coupled with high nitrogen (N_2_) fixing ability, has led the species to be regarded as a *“featured example of green manure’s contribution to the sustainable agricultural system”* [5].

Seeds of *M. pruriens* contain high levels (1–9%) of L-Dopa (L-3,4 dihydroxy phenylalanine) [14, 15] - a precursor of dopamine used in the treatment of Parkinson’s disease [16]. Daxenbichler et al. [17] screened 1000 species in 135 plant families and found only *Mucuna* spp. to contain sufficient L-Dopa for commercial use. Biochemically, L-Dopa is a non-protein amino acid produced as an intermediate product in the enzymatic synthesis of dopamine from L-tyrosine [18]. Although its efficacy for the treatment of Parkinson’s disease is widely recognized [19], ingestion of large amounts of *Mucuna* plant parts, particularly its nutritionally rich seeds, is discouraged due to potential toxicity and associated side effects from long-term consumption of L-Dopa [20]. Nonetheless, it is has been reported safe to consume 500 g/day of *Mucuna*-based food with L-Dopa content ≤ 0.1% [15].

Notwithstanding these benefits, the agronomic potential of *M. pruriens* has remained largely underexploited. Much needs to be done in terms of breeding efforts especially to develop improved varieties not only for high or low L-Dopa content, but also for enhanced nutritional value, resistances against biotic and abiotic stresses, and self-supporting, determinate cultivars. With the advent of genomic tools that can aid in developing molecular markers, genetic maps etc., the genetic improvement of underutilized crops has been greatly facilitated, enabling the development of improved genotypes or varieties with enhanced trait values [21, 22]. However, studies focusing on the development of genomic resources of *M. pruriens* are lacking, with only a few reports available on the use of molecular markers such as RAPDs and AFLPs (reviewed in [6]). In recent years, transcriptome sequencing has emerged as an efficient method to generate genomic-level data, large expressed sequence tag (EST) sequences, and molecular markers. Next generation sequencing (NGS) technologies are providing cutting-edge approaches for high-throughput sequence generation [22], allowing rapid and comprehensive analyses of plant genomes and cost-effective means of analyzing transcripts [23, 24]. Transcriptome sequencing has been successfully used for marker development in many underutilized legume plants, such as faba bean [25], adzuki bean [26], rice bean [27], hyacinth bean, grass pea and Bambara groundnut [28], and winged bean [28, 29], but has not yet been applied to research on *Mucuna*.

The present study reports the first transcriptome sequencing of *M. pruriens* genotypes. The objectives of this study were to (a) generate ESTs through whole transcriptome sequencing of two *M. pruriens* accessions; (b) develop and annotate a *de novo* transcriptome assembly; (c) discover and validate polymorphic microsatellite markers; (d) compare transcript expression in leaves, roots, and pods; and (e) perform genetic diversity and population structure analyses in a set of germplasm accessions for simple sequence repeat (SSR) marker validation. This study sets the stage for future molecular breeding, population and adaptation genomic studies, and provides a valuable resource for ongoing research into this agronomically and medicinally important legume species.

## Materials And Methods

### Plant material & RNA isolation

Two accessions representing different botanical varieties of *M. pruriens* and exhibiting contrasting phenotypes (Additional file 1: Table S1) were chosen for the study: *M. pruriens* var. *utilis* (IC0620620; collector’s ID: 500108KA) is a cultivated accession and *var. pruriens* (IC0620622; collector’s ID: 500113MH) is a wild accession. Accessions are available in the germplasm center of India. The plants were grown in the greenhouse facility of Sikkim University, Gangtok, India and a mapping population was developed. Young leaf, root and immature pod tissues were harvested for RNA isolation. Leaf, root, and pod tissues were chosen to maximize the number of genes expressed across tissues of different developmental processes and involved in key metabolic processes such as photosynthesis and respiration to 1) enable comparison with other transcriptomes utilizing the same tissue types (esp. Leaf) and 2) maximize sequencing across a range of developmental and metabolic processes.

Total RNA was isolated from each of the tissues using the method described by Ghawana et al. [30]. Pooled samples for both the accessions were prepared by combining equimolar concentration of total RNA for each of the tissues. Total RNA quality was assessed using NanoDrop-ND 2000C spectrophotometer and bioanalyzer. Samples with RIN (RNA integrity number) greater than 8.0 along with the ratios of 1.9-2.1 (260/280) and the ratios of 2.0-2.5 (260/230) were selected for sequencing.

### Synthesis of cDNA library and Illumina sequencing

RNA-seq library preparation and sequencing was carried out at Next Generation Genomics Facility (NGGF), Centre for Cellular and Molecular Platforms (C-CAMP), Bangalore, India. For tissues extracted from accession IC0620620, tissues were pooled prior to sequencing by combining equimolar concentration of total RNA for each of the tissues and sequenced as a single library. Four paired-end cDNA libraries were generated representing each tissue for IC0620622. The paired-end 2x100 bp library preparation was done following the protocol of the Illumina TruSeq RNA sample preparation kit (Illumina Inc.) as per the manufacturer’s instruction. One paired-end cDNA library was separately generated from the pooled RNA samples of genotype IC0620622. In total, five libraries were prepared and sequenced on a single lane of a 2×100 paired-end run by Illumina HiSeq^TM^ 1000.

### De novo assembly and redundancy removal

To obtain a robust overview of the transcripts in the *Mucuna* species, we generated a *de novo* assembly combining the filtered reads of the two accessions. Raw reads were filtered using quality value (Q) ≥30 and demultiplexed using an option of one mismatch in index. A total of 191 million reads from both the genotypes were used to develop the combined *de novo* assembly (referred to subsequently as 620–22). We used Trinity [31] with default parameters and a minimum contig length of 200 bp for assembly generation. To generate non-redundant transcripts, highly similar fragments were clustered using CD-HIT v. 4.6 [32] with 95% identity as cut-off, resulting in a total of 67,561 transcripts from the total of 72,561 Trinity assembled transcripts. Separate genotype-wise assembly was not performed at this stage due to the six-fold difference in the number of reads obtained between the two genotypes (1:6 relative ratios between IC0620620: IC0620622).

### Annotation and gene ontology

For protein functional annotation, transcripts longer than 200 bp were searched against non-redundant protein databases of NCBI, Swiss-Prot and Uniref90 using BLASTX with an E-value cut-off of 1e^-05^. BLASTX searches were also performed on the assembled transcripts using the legume database (http://plantgrn.noble.org/LegumeIP) and only the top hits were considered. The Annocript v1.1.2 pipeline [33] was employed to obtain Gene Ontology (GO) terms for describing biological process, cellular components, and molecular functions. The Enzyme Classes (EC) and Kyoto Encyclopedia of Genes and Genomes (KEGG) pathways were also annotated using the Annocript pipeline.

### Sequence similarity with other legume species

To compare the complement of genes characterized in the *M. pruriens* transcriptome assembly against gene assemblies in other legume species, protein sequences (*Medicago truncatula* Gaertn. Mt 4.0; *Glycine max* (L.) Merr. release 1.1; *Lotus japonicus* (Regel) K. Larsen release 2.5; *Phaseolus vulgaris* L. release 1.0; *Cicer arietinum* L. and *Cajanus cajan* (L.) Millsp. version 5.0) were downloaded from the NCBI database. BLASTX searches were performed on the 620–22 transcripts with an E-value cut-off of 1e^-05^, and the top hit for each transcript was used for further analysis.

### Mining transcription factor families

For mining transcription factor gene families, we downloaded the plant transcription factors database (PlnTFDB) 3.0 (http://plntfdb.bio.uni-potsdam.de/v3.0) [34] and queried *M. pruriens* transcripts against the PlnTFDB using BLASTX with an E-value cut-off of 1e^-05^.

### Mining of SSRs and detection of polymorphic SSRs

SSRs were searched in the 620-22 transcripts using the Perl script MISA (http://pgrc.ipk-gatersleben.de/misa) [35]. The microsatellite unit size and minimum number of repeats assigned was as follows: mono-nucleotide repeats more than 10 times, di-nucleotide repeats more than 6 times, tri-, tetra-, penta-, hexa-nucleotide repeats more than 5 times. The program was run up to deca- nucleotide repeats, but the results presented here are up to hexa- repeats only. For detecting the polymorphic SSRs between the mapping population parents, reads from each of the two genotypes were mapped to the 620-22 assembly. We used lobSTR v 3.0.3 [36] to identify polymorphic SSRs. This program provides a unique advantage over the conventional MISA pipeline as it can simultaneously compare two or more samples during the alignment process. We built a custom STR reference and the raw reads were passed through the program to be aligned around the SSR regions. BAM files from the alignment were sorted and indexed using samtools. Sorted BAM files were genotyped using the program allelotype within lobSTR. LobSTR identified 3,865 polymorphic SSRs, 787 of which were retained after selecting those having a quality value ≥10 and the alternative allele present in one of the samples. Of the 787 polymorphic SSRs, a subset of 134 SSRs was selected randomly and primers were designed using BatchPrimer3 (http://probes.pw.usda.gov/batchprimer3/) [37].

### Genic SSR amplification and validation

**Fig. 1:**
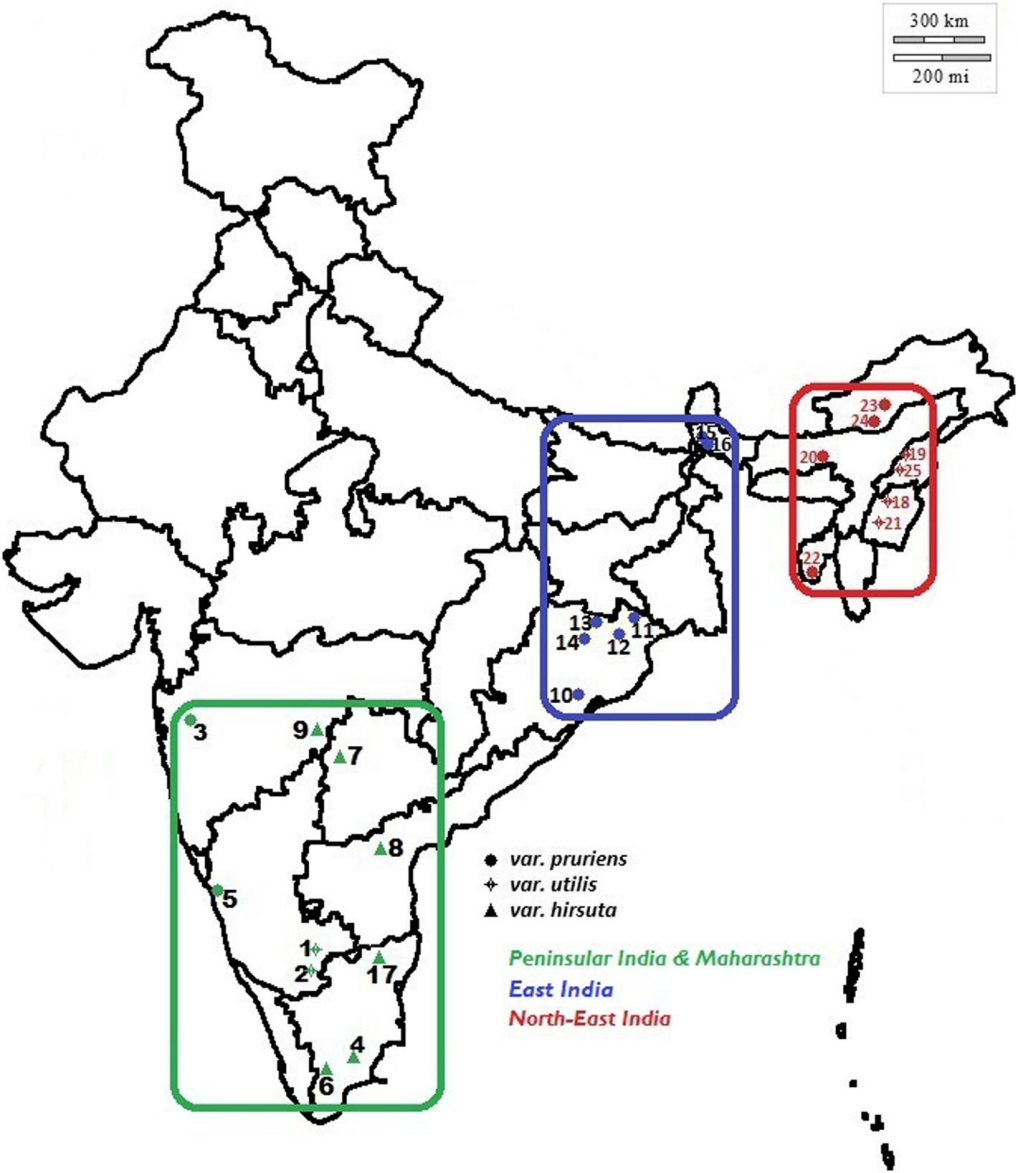
Map depicting collection locations of *Mucuna pruriens* used in this study

A total of 25 *M. pruriens* accessions (Table 1) representing different geographical locations in India (Fig. 1) were selected from the Sikkim University germplasm collection for marker validation using the 134 EST-SSRs. The collection comprised representative taxa from all the three botanical varieties of *M. pruriens* viz. var. *utilis* (*n* = 6), var. *pruriens* (*n* = 13), and var. *hirsuta* (*n* = 6). Genomic DNA was isolated from young leaves using a modified cetyltrimethylammonim bromide (CTAB) method [38]. PCR amplification was performed in a final volume of 25 μl containing 50 ng/μl of template DNA, 1X PCR buffer, 1.5 mM MgCl_2_, 2.5 mM dNTPs, primers (1 μM each) and 1U of Taq polymerase. The PCR conditions were as follows: initial denaturation at 94^o^C for 3 min followed by 35 cycles of 30s at 94^o^C, 30s at the annealing temperature (*Tm*) and 20s at 72^o^C with a final extension of 7 min at 72^o^C. The amplification was visualized using a UV illumination gel documentation system (Uvi-Tech DOL-008.XD, England). Subsequently, PCR products from different dye-labeled primers were pooled in equal volumes and 1.0 μl each of amplicons were mixed with 7 μl of formamide, 0.05 μl of the GeneScan™ 500 LIZ® Size Standard (Applied Biosystems, USA) and 2.95 μl of distilled water. DNA fragments were denatured and size fractioned using capillary electrophoresis on an ABI 3730 DNA Genetic Analyzer (Applied Biosystems, USA).

**Table 1:** Details of accessions used for EST-SSR validation

Sample Number	Accession number/Collector ID	Variety	Latitude (N)	Longitude (E)	Altitude (AMSL) (m)	State of Origin
1	500101KA*	var*. utilis*	13°14′	77°62′	911	Karnataka
2	IC0620620**	var*. utilis*	13°14′	77°62′	911	Karnataka
3	IC0620622**	var*. pruriens*	20°00′	73°77′	745	Maharashtra
4	500120TN*	var*. hirsuta*	9°55′	78°07′	138	Tamil Nadu
5	IC0620624**	var*. pruriens*	14°48′	74°12′	7	Karnataka
6	500136TN*	var*. hirsuta*	10°04′	77°45′	298	Tamil Nadu
7	500147AP*	var*. hirsuta*	18°39′	78°10′	383	Telangana
8	500154AP*	var*. hirsuta*	16°04′	78°52′	434	Andhra Pradesh
9	500186MH*	var*. hirsuta*	19°09′	77°27′	373	Maharashtra
10	500192OR*	var*. pruriens*	20°18′	85°62′	63	Odisha
11	500193OR*	var*. pruriens*	21°94′	86°72′	51	Odisha
12	500194OR*	var*. pruriens*	21°94′	86°72′	51	Odisha
13	500195OR*	var*. pruriens*	21°63′	85°58′	650	Odisha
14	500196OR*	var*. pruriens*	20°47′	85°12′	121	Odisha
15	500197WB*	var*. pruriens*	26°71′	88°43′	125	West Bengal
16	500199WB*	var*. pruriens*	26°70′	88°80′	65	West Bengal
17	500202TN*	var*. hirsuta*	12°57′	79°56′	43	Tamil Nadu
18	500210MN*	var*. utilis*	25°41′	94°47′	782	Manipur
19	500211NL*	var*. utilis*	25°67′	94°12′	1333	Nagaland
20	500212AS*	var*. pruriens*	26°11′	91°44′	61	Assam
21	500217MN*	var*. utilis*	25°68′	93°03′	776	Manipur
22	500219TR*	var*. pruriens*	23°50′	91°25′	64	Tripura
23	500221AR*	var*. pruriens*	27°08′	93°40′	1035	Arunachal Pradesh
24	500224AR*	var*. pruriens*	27°08′	93°40′	296	Arunachal Pradesh
25	500267NL*	var*. utilis*	25°68′	94°08′	1360	Nagaland

*Collectors ID of newly collected accessions; **National genebank ID

We applied stringent filtering criteria (minor allele frequency > 0.05 and missing percentage less than 20% for each marker band) for the marker bands produced, which resulted in 52 primer-pairs and 125 high-quality alleles/bands for all the downstream analysis. For data analysis, each marker-band was scored as a dominant marker with presence of a band in a genotype indicated as “1” and absence “0.” Further, the genotyping data were converted into bi-allelic format (e.g., 1 denoted as AA and 0 as GG) and a hapmap file was generated. The hapmap file was converted into Variant Call Format (VCF) using TASSEL v 5.2.29 [39]. The quality control on the dataset was performed using VCFtools [40]. Markers with minor allele frequency (MAF) >0.05, max-missing sites <20%, and accessions with maximum missing site <20% were retained. Estimates of expected heterozygosity (He), observed heterozygosity (Ho), effective number of alleles (Ne) [41], polymorphism information content (PIC), gene diversity (h) [42], and Shannon’s Information index (I) [43] were calculated using the software GenAlex 6.5 [44] and POPGENE [45]. Phylogenetic tree construction using the neighbor-joining (NJ) algorithm and principal component analysis (PCA) were performed using TASSEL. The resulting tree was visualized as a mid-point rooted tree using FigTree v1.4.2 [46]. A plot of PC1 versus PC2 was made in R (http://www.r-project.org/) [47] using the ggplot2 package (ggplot2.org) and the geom_text_repel function in ggrepel (http://github.com/slowkow/ggrepel) to plot accession names. Population structure was investigated using a Bayesian framework implemented in the program fastSTRUCTURE [48] with the following commands: --prior simple –full –seed = 100 –cv = 5 for subgroups 2 to 10 (K = 2 to 10). The output was investigated with “choosing model complexity” script included in the program fastSTRUCTURE and by plotting marginal likelihood and cross-validation error against the number of subgroups to determine the possible range of subgroups. The range of subgroups identified were then inspected with proportion of membership of genotypes to respective subgroups (coefficient of ancestry values) and known geographical origin information to determine the precise number of subgroups in the collection. The results were visualized using a plot made in R with ggplot2, reshape (http://had.co.nz/reshape) and RColorBrewer (http://colorbrewer2.org) packages.

### Expression analysis

For analyzing differentially expressed transcripts, raw reads obtained from transcriptome sequencing of leaf, root, and pod tissues of the *M. pruriens* var. *utilis* (IC0620620) were aligned separately to the 620-22 assembly using bowtie aligner version 1.1.1 [49]. We normalized the gene expression level in each library to produce an effective library size for use in calculating read counts. We used RSEM version 3.0 [50] to calculate the read count and the estimated expression levels as fragments per transcript kilobase per million fragments mapped (FPKM) using edgeR software in R [51]. A dispersion value of 0.1 was used in the expression analysis. Differentially expressed transcripts (DETs) were determined with log-fold expression change ≥4 and a statistically significant p-value of 0.001. Pair-wise comparisons among the three tissues were conducted by comparing the sequenced samples to identify common DETs across all the tissue types. The top fifty DETs from pair-wise comparisons across tissues were extracted to generate a heat map and demonstrate the dynamic expression patterns in different tissues. In addition, we also extracted the top differentially expressed transcripts from secondary metabolite biosynthesis classes based on annotation to determine expression patterns related to secondary metabolite pathways.

## Results

### Sequencing and de novo assembly

Illumina sequencing generated 18.24 GB of data containing 167,986,452 and 27,801,324 raw reads for genotypes IC0620620 and IC0620622, respectively (Table 2). The combined assembly 620-22 produced 72,561 transcripts. After clustering, 67,561 transcripts were retained with an N50 length of 987 bp and a mean transcript length of 641 bp (Table 3; Additional file 1: Figure S1).

**Table 2:** Summary of data generated for *Mucuna pruriens* transcriptome. G1 is *Mucuna pruriens* var. *utilis* (IC0620620; collector’s ID: 500108KA); G2 is *M. pruriens var. pruriens*(IC0620622; collector’s ID: 500113MH)

Sample	fastq file size (GB)	Total number of paired end reads	Total number of reads after quality filtering
G1 Leaf	1.86	19,406,426	18,997,424
G1 Pod	5.42	58,585,008	57,166,422
G1 Root	2.69	28,623,354	28,046,508
G1 Pooled	5.68	61,341,664	59,885,295
G2 Pooled	2.59	27,801,324	27,137,593
Total	18.24	195,757,776	191,233,242

**Table 3:** Statistics of non-redundant set of *Mucuna pruriens* transcripts obtained from Trinity assembly

Total number of assembled bases	46,525,999
Number of transcripts	72,561
The total number of transcripts after clustering	67,561
The mean sequence length	626
Average % of N	0.00
Average % of GC content	44.58
N50	987
Maximum transcript length	17,978
Average transcript length	641
Number of putative non coding sequences	1,493
Length of the longest ORF (bp)	2,362
Number of ORFs ≥ 100 bp	36,228
Number of ORFs on plus (+) strand	36,421
Number of ORFs on minus (-) strand	31,140

### Functional annotation and characterization of *M. pruriens* transcripts

**Fig. 2:**
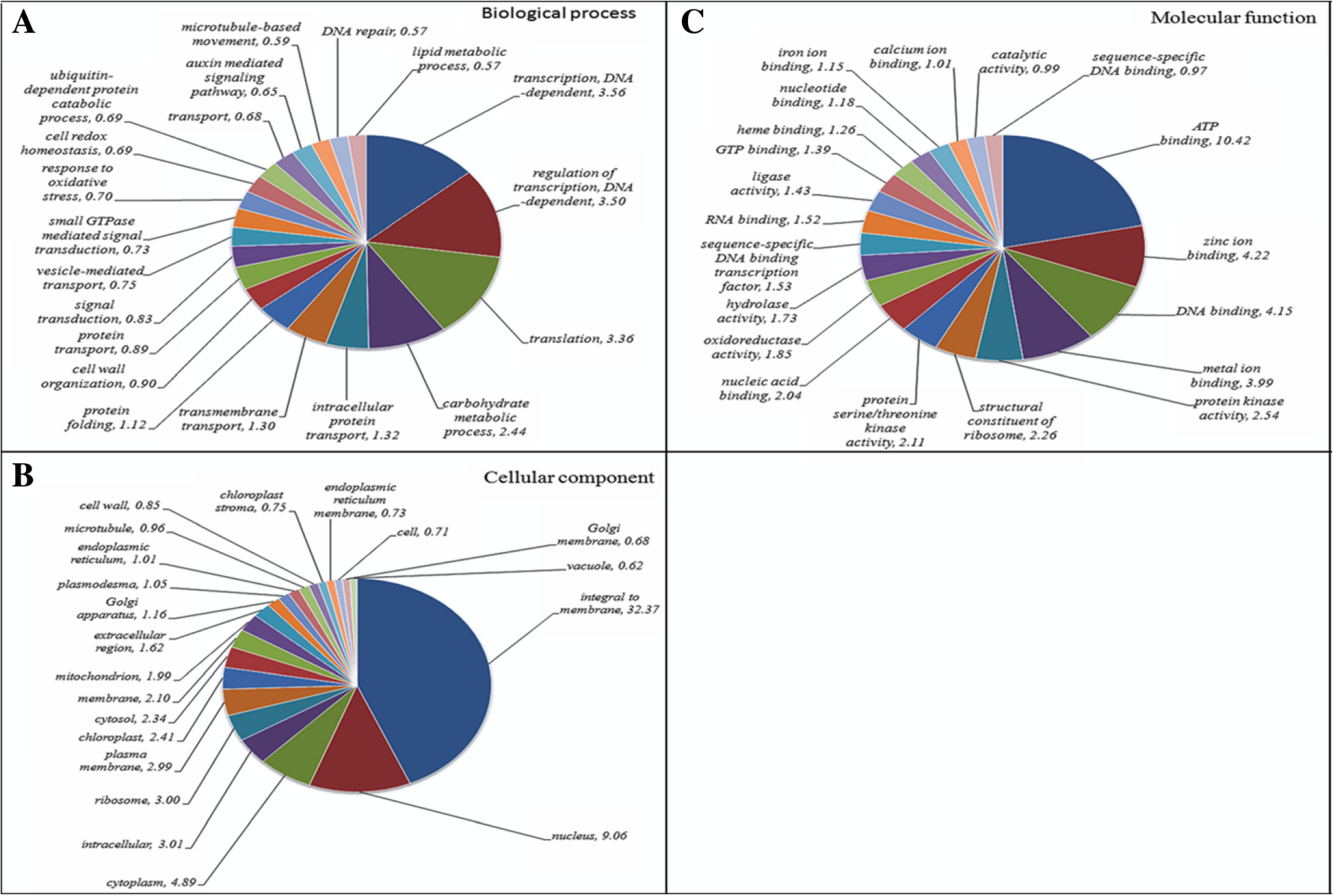
Functional annotation of Mucuna pruriens transcripts. Gene ontology term assignments to transcripts in different categories of **a** biological process, **b** cellular component, and **c** molecular function. Numbers are percentage of function for each major category

A total of 49,925 (73.9%), 35,535 (52.6%) and 54,450 (80.6%) transcripts showed significant hits with NCBI-NR, Swiss-Prot and UniRef proteins, respectively, with 34,686 transcripts having conserved domains and 6,248 with hits against the Rfam database. Broadly, the putative orthologs of genes involved in various pathways and cellular processes were found to be conserved in *M. pruriens*. Further, GO terms were assigned to *M. pruriens* transcripts that showed significant similarity with annotated proteins from other plant species (Additional file 2). A total of 30,575 (45.3%) transcripts were assigned at least one GO term in the biological process category, 46,961 (69.51%) in the molecular function category and 30,199 (44.70%) in the cellular component category. Among the various biological processes, genes coding for proteins involved in transcription (3.56%) and transcription regulation (3.50%) were highly represented. The genes involved in other important biological processes such as carbohydrate metabolism, signal transduction, response to stress, transport, cell wall organization and protein folding were also identified through GO annotations. Similarly, ATP, DNA as well as different metal ion binding activities were most represented among the molecular functions; and integral membrane, nucleus and cytoplasm related activities were most represented among the cellular component categories (Fig. 2).

Enzyme classes were obtained for 3,963 assembled sequences, whereas associated KEGG classification was obtained for 3,492 assembled sequences (Additional file 3). The top 20 abundant enzyme classes observed for the *M. pruriens* transcriptome are listed in Additional file 1: Figure S2A. The greatest number of assembled transcripts belonged to the serine/threonine protein kinase enzyme class (38.4%). Besides this, Additional file 1: Figure S2B displays the top 20 KEGG pathways represented by the assembled transcriptome sequences. The highest number of sequences belonged to protein modification pathways (37.5%) followed by lipid metabolism and glycan metabolism. As evident from the results, the highest represented groups included several pathways associated with housekeeping processes as well as plant development and secondary metabolism.

### Sequence similarity with other legume species

A comparison of assembled transcripts against proteomes of chickpea, pigeon pea, soybean, common bean, mung bean, garden pea, barrel medic and Lotus showed that 58,208 of 67,561 transcripts (86.2%) from the 620–22 assembly had significant similarity to sequences in one or more legumes (Additional file 4). About 71% of these transcripts had ≥ 70% sequence identity (Additional file 1: Figure S3A). The largest number of *M. pruriens* transcripts showed significant similarity with soybean transcripts followed by *Medicago*, *Phaseolus, Vigna*, *Cicer* and the least similarity with *Pisum* and *Cajanus* (Additional file 1: Figure S3B). The lack of strong correlation with taxonomy (e.g. greater similarity to *Medicago* than to the phaseoloid, *Cajanus*) is presumably due to varying quality of the different genome assemblies.

### Mining transcription factor families

**Fig. 3:**
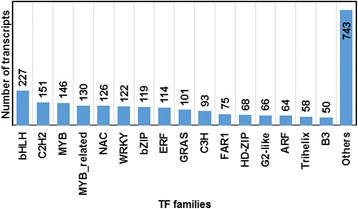
Distribution of *Mucuna pruriens* transcripts in different transcription factor families

In total, 2,223 putative *M. pruriens* transcription factors distributed in at least 55 families were identified representing 3.29% of *M. pruriens* assembled transcripts (Additional file 5). Among these, the basic/helix-loop-helix (bHLH; 227), C2H2-type (151), MYB (146), MYB related (130), NAC (126) and WRKY (122) were among the top categories (Fig. 3). However, almost all the families showed minor species-specific differences in relation to TF gene families reported for *Cicer*, *Lotus*, *Medicago* and *Glycine* (Table 4)*.*

**Table 4:** Number of transcripts encoding for transcription factor families in *Mucuna pruriens* compared to other legumes*.* The data on *M. pruriens* is from our study; data for soybean, *Medicago* and *Lotus* is from Libault *et al* [69]; data for Chickpea is from Garg *et al* [70]

TF family	*M. pruriens*	Chickpea	Soybean	*Medicago*	*Lotus*
bHLH	227	488	393	71	64
AUX/IAA-ARF	64	216	129	24	36
C2C2-CO-like	16	15	72	15	21
C2C2-GATA	44	49	62	29	16
C2C2-YABBY	13	8	18	6	4
C3H	93	594	147	41	50
CAMTA	18	26	15	6	4
MYB	146	528	791	171	191
PHD	10	489	222	45	47

### Detection of genic SSR markers

**Fig. 4:**
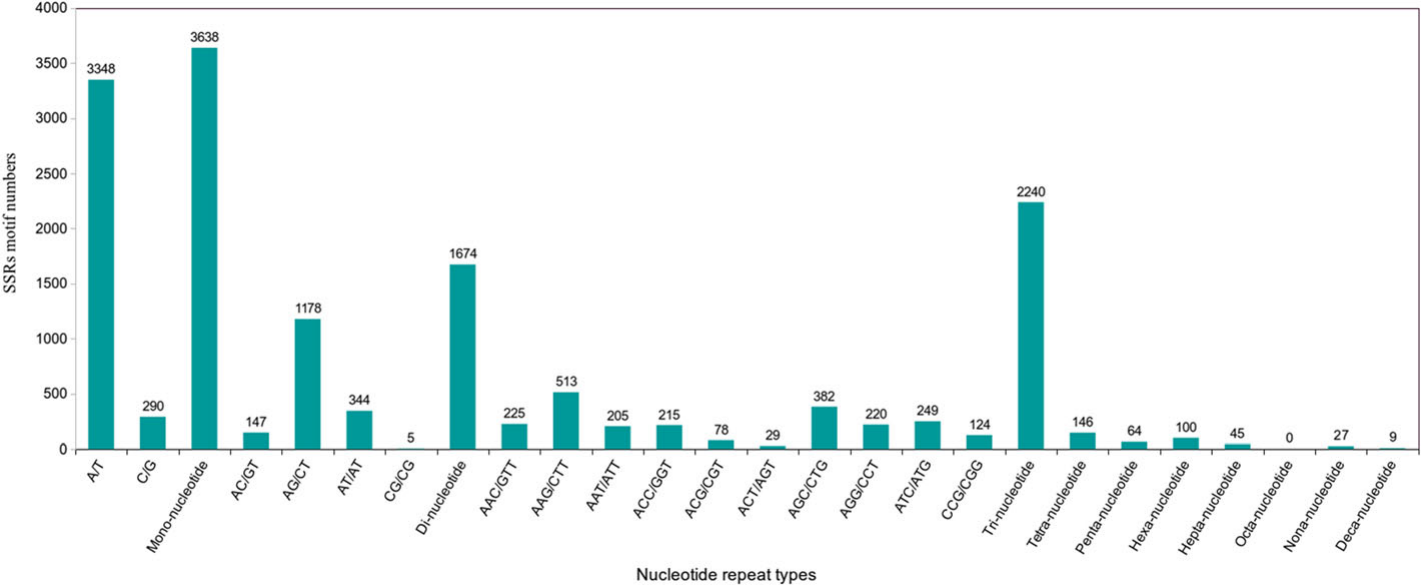
Simple sequence repeat length distribution across different motif classes in *Mucuna pruriens* transcriptome

We detected a total of 6,284 transcripts (Additional file 6) within which 7,943 potential EST-SSRs (Additional file 7) were discovered. The mono-nucleotide SSRs represented the largest fraction (3,638), with the vast majority (92%) comprising A or T repeats, which likely represent remnants of mRNA poly-A tails (Fig. 4). Only a small fraction of tetra-nucleotide (146), penta- (64) and hexa- (100) nucleotide SSRs were identified in *M. pruriens* transcripts (Table 5). Of the 6,284 SSR-containing sequences, 1,174 transcripts contained more than one SSR. Further, EST-SSRs with five tandem repeats were most common, followed by ten, six, eleven, seven, and twelve tandem repeats, whereas the remaining tandem repeats each accounted for less than 5% of the our EST-SSRs (Additional file 1: Table S2).

**Table 5:** Statistics of SSRs identified in *Mucuna pruriens* transcripts

SSRs mining	
Total number of sequences examined	67,561
Total size of examined sequences (bp)	42,340,968
Total number of identified SSRs	7,943
Number of SSR containing sequences	6,284 (9.3%)
Number of sequences containing more than one SSR	1,174
Number of SSRs present in compound formation	963
Frequency of SSRs	One per 5.3 kb
Distribution of SSRs in different repeat types	
Mono-nucleotide	3,638 (45.80%)
Di-nucleotide	1,674 (21.07%)
Tri-nucleotide	2,240 (28.20%)
Tetra-nucleotide	146 (1.83%)
Penta-nucleotide	64 (0.80%)
Hexa-nucleotide	100 (1.25%)

Screening for SSRs using lobSTR identified a total of 3,865 SSRs polymorphic between the parents (Additional file 8). Aligning IC0620622 (G2) reads against the combined assembly yielded 3,075 SSR calls, with 1,339 of these present in areas with greater than 5x coverage and a mean coverage of 9.54x. Alternatively, alignment of IC0620620 (G1; 500108) reads to the combined assembly yielded 3,517 SSR calls, 2,092 of which were present in areas with greater than 5x coverage and with a mean coverage of 19.15x. After filtering the SSRs based on the parameters mentioned earlier, we obtained a total of 787 polymorphic repeats (Additional file 8). The details of the SSR repeats from the lobSTR analysis are given in Additional file 1: Table S3 and the distribution of different SSR motifs from the lobSTR output is presented in Additional file 1: Figure S4.

### EST-SSR validation and population structure

Of the 134 primer pairs selected, 98 (73.13%) successfully amplified the genomic DNA of 25 *M. pruriens* accessions. From this, consistently amplified marker-bands from 82 primer pairs were chosen for further analysis. Nearly 2,000 marker bands were amplified by these 82 primer pairs and 125 high-quality marker-bands representing 52 primer pairs across 23 accessions were retained after quality control (Additional file 1:Table S4). Accessions 500267NL and 500101KA were dropped due to >20% missing marker information. Various measures of genetic diversity for each primer pair are reported in Additional file 1: Table S4. Parameters of genetic diversity were also estimated between the population groups representing different geographical locations (3) and botanical varieties (3). The genetic diversity index (h) between different geographical locations and botanical varieties ranged from 0.35 to 0.37 and 0.34 to 0.36 with mean values of 0.35 and 0.35 respectively (Table 6), whereas h was much lower for genetic subgroups, ranging from 0.16 to 0.21. In all groups, the total gene diversity, Ht, was higher than the gene diversity within the groups, Hs. The coefficient of gene differentiation (Gst) was 0.04, which indicated very less genetic differentiation among different population groups as compared to within group variations. Gene flow indices (Nm) were relatively high, ranging from 1.83 to 4.09.

**Table 6:** Gene diversity estimates for groups based on botanical varieties, geographical distribution and population structure analysis

	Population group	Na	Ne	I	h
Geographical distribution	East India	2.23	1.78	0.59	0.37
	North East India	2.21	1.68	0.52	0.36
	Peninsular India	2.98	1.95	0.72	0.35
		Ht	Hs	Gst	Nm
	Mean	0.41	0.36	0.04	4.09
	SD (±)	0.19	0.18		
Botanical varieties	var. *pruriens*	2.67	1.83	0.64	0.36
	var. *hirsuta*	2.46	1.78	0.60	0.36
	var. *utilis*	2.10	1.84	0.59	0.34
		Ht	Hs	Gst	Nm
	Mean	0.43	0.36	0.04	2.57
	SD (±)	0.19	0.17		
Population groups based on K = 4 sub grouping	SG1	2.54	1.91	0.66	0.19
	SG2	1.87	1.53	0.41	0.16
	SG3	2.36	1.77	0.59	0.21
	SG4	2.10	1.84	0.60	0.18
		Ht	Hs	Gst	Nm
	Mean	0.41	0.34	0.04	1.83
	SD (±)	0.19	0.17		

Na- Number of alleles; Ne- Effective no. of alleles [41]; I- Shannon information content; h- Nei’s gene diversity [42]

**Fig. 5:**
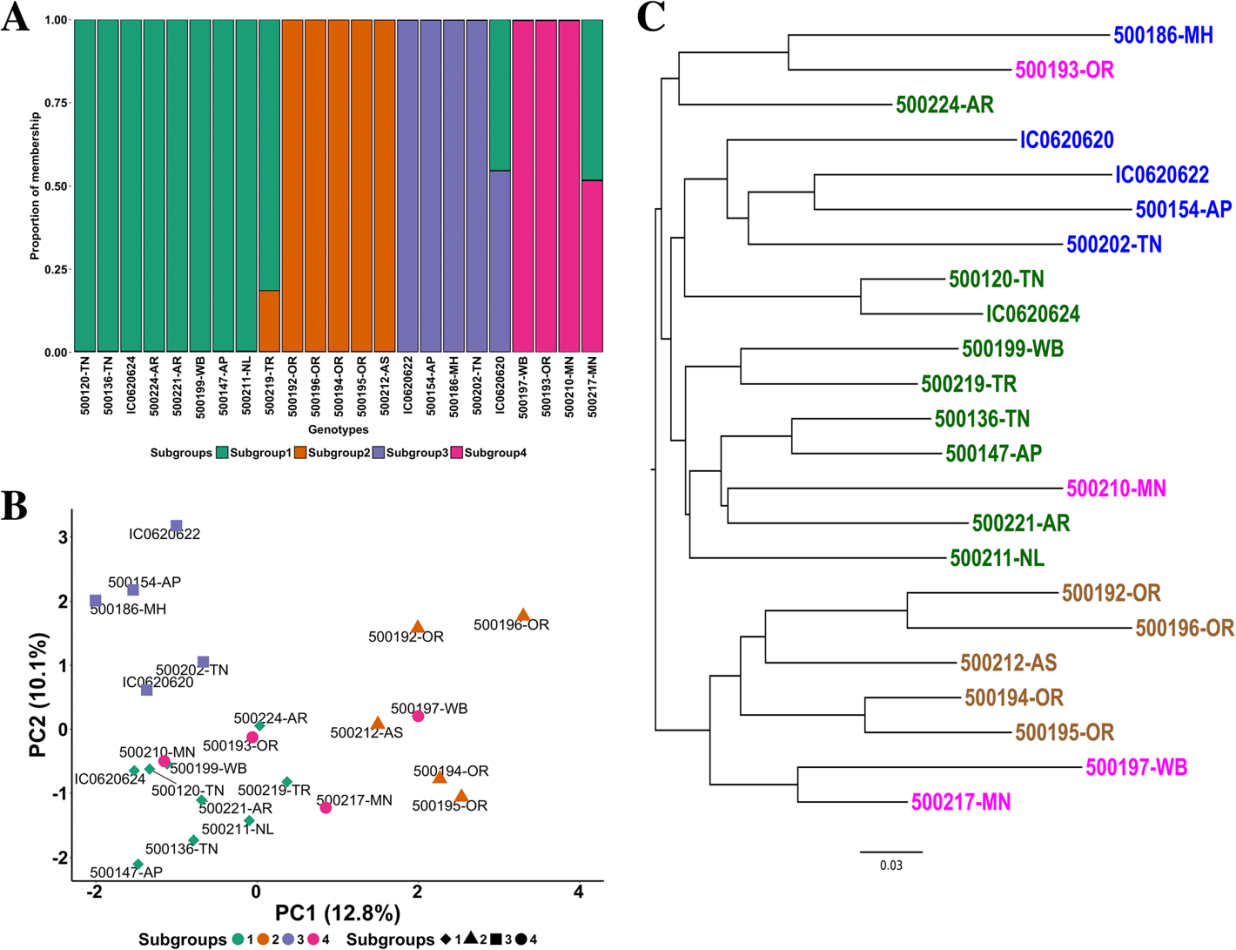
Population Structure analysis of the 23 Indian *Mucuna pruriens* accessions. **a** Bayesian clustering (fastSTRUCTURE, *K = 4*); **b** Scatter plot from principal component analysis (PCA); **c** Neighbor-joining tree generated for all accessions

Population structure analysis using fastSTRUCTURE on the 23 genotypes suggested the presence of 4 to 6 subgroups using the “choosing model complexity” script, and 4 or 8 subgroups based on likelihood score (Additional file 1: Figure S5), with K = 4 being the most probable. For K = 4 subgroups, 21 genotypes had >80% proportion of membership to a respective subgroup as determined by the coefficient of coancestry value of each genotype (Fig. 5a). Subgroup 1 is composed of nine individuals, mostly of var. *pruriens* (*n* = 5) and only one var. *utilis*, with mixed representation from peninsular and northeast India (each *n* = 4) but only a single accession hailing from eastern India. Subgroup 2 is exclusively composed of var. *pruriens* from eastern (*n* = 4) and northeastern (*n* = 1) India. Subgroup 3 is exclusively from peninsular India with most accessions of classified as var. *hirsuta* (*n* = 3), but with one each from the other varieties. Subgroup 4 is the most heterogeneous group and includes two var. *pruriens* accessions from eastern India and two var. *utilis* accessions from northeast India.

The genotypes in the NJ tree and PCA are color coded from the information of 4 subgroups in the fastSTRUCTURE. NJ and PCA revealed similar clustering of genotypes as fastSTRUCTURE with at least three out of the four groups clustering largely according to their geographical origin (Fig. 5). Principal component 1 (PC1; Fig. 5b) accounted for 12.8% of genetic variability and separated the majority of northeastern and eastern accessions from peninsular + Maharashtra accessions, with five northeastern or eastern accessions falling on the left side. No peninsular accessions fell right of PC1. Furthermore, all *hirsuta* and all *utilis* accessions but one were found left of PC1. The *utilis* accession (500217MN, northeast India) is one of two individuals that could be interpreted as having a hybrid ancestry, evidenced by a ~50% split assignment to subgroup1 and subgroup 4, the other accession being IC0620620 (var. *utilis*, peninsular India) and assigned to subgroups 1 and 3 with near equal probability. Subgroup 2 is entirely separated from subgroup 3 by PC1. Principal component 2 (PC2; Fig. 5b) accounted for another 10.1% of genetic variability. Subgroup 1, the largest and most diverse subgroup, clustered exclusively below PC2.

The neighbor joining algorithm produced two main clades (Fig. 5c), one equating to a similar grouping separated in PCA by PC1 which is also the same as subgroup 2 plus two accessions from subgroup 4. This clade includes only var. *pruriens* from the northeastern and eastern areas with the exception of the potential hybrid 500217MN, var. *utilis*. The second clade includes subgroups 1 and 3 plus two accessions of subgroup two, with no clear clustering based on variety.

### Expression analysis

**Fig. 6:**
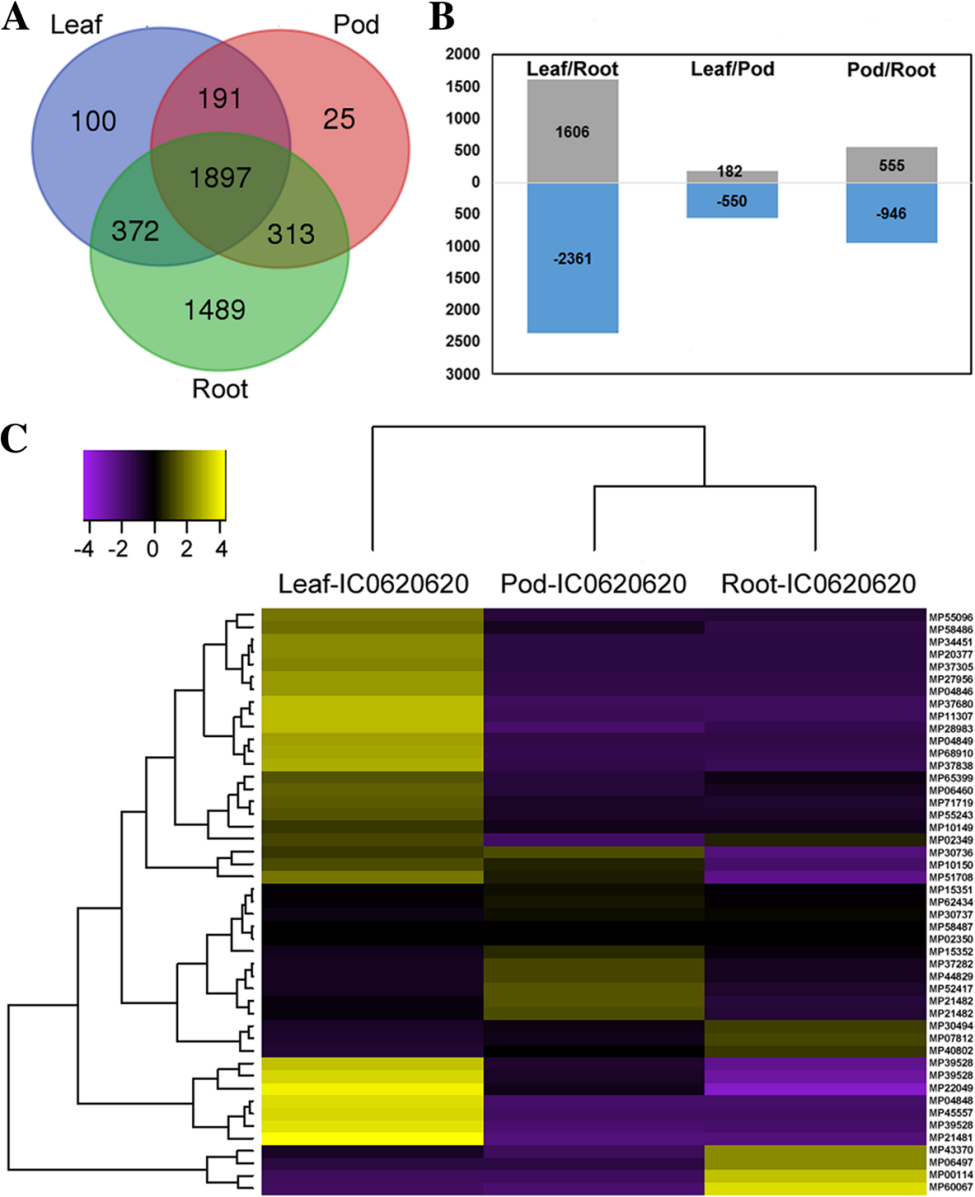
Differential transcript expression in leaf, root, and pod tissues. **a** Diagram showing overlap of genes between leaf, root, and pod tissues showing differential transcript expression. **b** Pairwise comparisons across tissues showing differentially expressed transcripts. Those above the line are transcripts up-regulated and those below are down-regulated within the pairwise comparison. **c** Heat map of secondary metabolite associated differentially expressed genes of leaf, pod, and root transcriptomes. The various shades in the boxes showed similar tendencies of gene expression. Labels along the right side correspond to transcript names (see Additional file 9)

A total of 4,387 transcripts were differentially expressed among three tissues of IC0620620 with log-fold expression change ≥4 and a statistically significant p-value of 0.001, of which 1,897 were commonly expressed in all three tissue types; 191 to 372 were shared between tissue types and 25 to 1489 were unique to tissues, respectively (Fig. 6a). In leaf, 1,606 transcripts exhibited up-regulation and 2,361 transcripts exhibited down regulation as compared to roots, followed by 182 up-regulated and 550 down-regulated against the pod tissue. Similarly, pairwise comparison of pods and roots exhibited 555 and 946 transcripts were up-and down- regulated respectively (Fig. 6b). The top fifty differentially expressed transcripts in each of the three tissues are visualized to show varying expression patterns (Additional file 1: Figure S6). Among the differentially expressed transcripts, 223 were found to encode for TFs representing 43 different families, including MYB, MADS, WRKY, and bHLH families, some of whose members are involved in secondary metabolite biosynthesis. We also investigated the expression of other genes involved in secondary metabolism/biosynthesis and observed that the top 47 transcripts in this category showed varying differential expression patterns (Fig. 6c). Among these, highly expressed transcripts included those belonging to flavonoid, isoprenoid, phenylpropanoid, and wax pathways (Additional file 9).

## Discussion

### Developing genetic resources for *Mucuna* through transcriptomics

The legume family is second only to the grass family (Poaceae) in economic importance, with a number of species utilized as crops, fodder, industrial agents, construction materials, and medicines [52]. The genomes of legume species including soybean [53], the common bean (*Phaseolus vulgaris* L.) [54], cowpea (*Vigna unguiculata* (L.) Walp.) [55], *Medicago sativa* L. [56], *Lotus japonicus* L. [57], pigeonpea (*Cajanus cajan* (L.) Millsp.) [58], and Lupin (*Lupinus angustifolius* L.) [59] have been sequenced, providing critical genomic resources across Fabaceae. However, a number of economically important legumes, including orphan crop species or those used for purposes other than human consumption, are in need of efforts to develop genetic and genomic resources to act as intellectual infrastructure upon which to build a broader scientific future. To our knowledge, we present the first *de novo* transcriptome assembly described for *Mucuna pruriens*, the velvet bean, an orphan legume crop used both for human consumption and medicine, providing an important genetic resource base for future genetic studies and breeding efforts.

*Mucuna* is a monophyletic genus [60] that represents an early-branching, distinct evolutionary lineage within the phaseoloid legumes. *Mucuna* is variously allied with monotypic *Haymondia wallichii* (DC.) A.N.Egan & B.Pan bis [4, 61] or with tribe Desmodieae [4, 62–64]. The addition of our transcriptomic data representing the *Mucuna* lineage thus fills a void within the developing resource base of transcriptomic data available for comparative evolutionary studies across legumes.

Within our species-level transcriptome assembly, we recovered 67,561 transcripts and annotated over 86% of these against one or more legume proteomes (Additional file 1: Figure S3), presenting a large collection of expressed genes that can be used in downstream analyses for genetic study and crop improvement programs. Our total number of transcripts recovered is quantitatively similar to a number of recent legume transcriptomic studies. As an example, Ištvánek et al. [65] recovered 64,761 transcripts from red clover (*Trifolium pratense* L.), ~73% of which were annotated. From a genomic perspective, our complement of annotated genes may seem high for a diploid species. The common bean, *Phaseolus vulgaris* L., is estimated to have ~28,000 coding genes [54], Chinese licorice, *Glycyrrhiza uralensis* Fisch., has ~ 35,500 genes [66], whereas soybean, *Glycine max* (L.) Merr., a recent polyploid crop [67], has between ~46,000 and 56,000 protein-coding genes (*Glycine max* Wm82.a2.v1 build at phytozome.jgi.doe.gov; [53]). The higher number of annotated transcripts in our study may be due to the presence of multiple isoforms, alternatively transcribed transcripts, and/or portions of genes that did not completely assemble with the settings we used. Our higher number of annotated genes could also include an increased complement of coding genes necessary for plants that produce a high degree of secondary metabolites [68].

Transcription factors (TFs) play important roles in gene regulation and function. We assessed the number and distribution of TF gene families within *M. pruriens* (Fig. 3; Table 4) and found that the overall distribution of transcription factor encoding transcripts among the various known protein families is similar to that of soybean and other legumes [69]. Transcription factors constituted ~3.3% of annotated transcripts, a number similar to that in *Medicago truncatula* and *Lotus japonicus* but slightly higher than that of *Psophocarpus tetragonolobus* (Table 4, Additional file 5; [29]) and considerably lower than that estimated for soybean [54] or chickpea [70]. Soybean likely has a higher number of TFs than most legumes due polyploidy. The MYB and basic-Helix-Loop-Helix (bHLH) TF gene families are two of the most prevalent TF gene families in our data, both of which play important roles in secondary metabolite biosynthesis, particularly for flavonoid and anthocyanin compounds [71]. In terms of differences within TF gene families of *Mucuna* relative to other legumes, we noted several events of expansion (e.g. C2C2-GATA, CAMTA) and contraction (e.g. PHD), evidence of the evolving nature of TF across lineages within legumes.

### Differential transcript expression across tissues within *Mucuna*

Differential transcript expression analysis performed by pair-wise comparison among the three tissues of genotype IC0620620 (Fig. 6 and Additional file 9) found thousands of differentially expressed transcripts across leaves, roots, or pods. Transcripts highly expressed in one tissue versus others suggest tissue-preferred expression, which can be helpful for further studies (Additional file 1: Figure S6). For example, transcripts involved in anthocyanin biosynthesis were upregulated in leaves relative to pods or roots (Fig. 6c). Uniquely expressed genes in leaf, root or pod tissues will be of importance to understand their contributions towards economically important traits in *Mucuna*. Genes involved in secondary metabolism are especially important as *Mucuna* is a natural source of mucunain and serotonin, chemicals found in pod hairs that promote itching [72], and of high levels of L-dopa found in seeds [73]. L-dopa is the precursor to dopamine, norepinephrine, and epinephrine (adrenaline), important neurotransmitters in the brain, and is widely used in the treatment of Parkinson’s disease [74]. Recent studies have shown that some Parkinson’s patients better tolerate taking ground *Mucuna* seeds as a source of L-DOPA and that this natural source may be more effective and neuroprotective than L-DOPA itself while lessening adverse side effects [19]. We have initiated further investigation on the expression of genes specific to L-Dopa and other important secondary metabolites in this plant to gain further understanding on the regulation of genes involved, the results of which will be reported in a secondary paper.

Tissue-preferred expression of transcripts involved in secondary metabolism has been found in other plants, especially those used for medicinal purposes (e.g. in citronella, *Cymbopogon winterianus* Jowitt [75]). Beyond L-Dopa, many other important secondary compounds have evolved within legumes [76]. For comparisons between root and leaf, those transcripts upregulated in the root relative to leaf transcriptomes were mostly related to secondary metabolism. A similar result was found for comparison of root and shoot tissues in *Leucaena leucocephala* (Lam.) de Wit [77]. In our study, the most highly upregulated gene involved in secondary metabolism in the root transcriptome was isoflavone reductase which exhibits a ~6-fold increase relative to pods and leaves (unigene MP60067 in Fig. 6c and Additional file 9). Isoflavone reductase is an enzyme unique to the plant kingdom involved in the isoflavonoid phytoalexin biosynthesis pathway and is suggested to play important roles in stress responses. Overexpression of isoflavone reductase in soybean was shown to enhance resistance to the oomycete *Phytophthora sojae* and induce antioxidant activity in the plant [78]. As root secondary metabolites have been less investigated in *M. pruriens,* this work may enable new areas of research and lead to discovery of novel secondary compounds of pharmaceutical interest.

Other transcripts related to secondary metabolites, such as anthocyanins, showed differential expression in leaf tissue relative to pods and roots. These plant pigments produce dark colors, particularly blue and purple, in above-ground plant tissues and also provide important antioxidant properties [79]. The strongest secondary metabolism-related transcript upregulated in the leaves was chalcone synthase, the first step in the phenylpropanoid pathway that leads to production of many flavonoid secondary metabolites, including anthocyanins [80]. NAD(P)H-dependent 6’ deoxychalcone synthase was moderately upregulated in the pod transcriptome and is an enzyme involved in synthesis of isoliquiritigenin, a secondary compound known primarily from licorice (*Glycyrrhiza spp.*), a related legume genus. Isoliquiritigenin provides a number of useful pharmacological properties such as anti-inflammatory, anti-viral, anti-microbial, and cardioprotective effects and has shown remarkable anti-cancer properties [81]. In combination with other transcriptomic and genomic resources, our transcriptome provides a useful resource for genetic studies related to secondary metabolites of medicinal application and interest.

### Detection and validation of microsatellite (SSR) markers in *Mucuna pruriens*

The medicinal potential afforded by secondary chemistry within *Mucuna* has no doubt led to its popularity throughout India where it is a component in over 200 indigenous Ayurvedic drug formulations used against a wide range of disorders, such as menstrual discomfort, neurological issues, sexual dysfunctions, tuberculosis and even elephantiasis [14]. Consequently, *M. pruriens* is found throughout India in both cultivated and wild forms. Microsatellites, or SSRs, are excellent genetic markers to aid in construction of genetic linkage maps and association analysis. As such, development of a database of SSR markers known to be polymorphic within *M. pruriens* may be useful for future genetic improvements of this important medicinal plant. Within our transcriptome, we detected over 4,000 EST-SSRs of di-nucleotide or higher repeats (Table 5; Fig. 4). Of these, tri-nucleotide repeats were the most abundant, a sensible result given the coding nature of the transcriptome [82]. Other legumes exhibit the same trend, including the winged bean [29] and peanut [83].

Certain repeat motifs were more prevalent than others in our ~3,800 EST-SSRs polymorphic between parents (summarized in Additional file 1: Table S3), a finding noted in other legumes previously [29, 84]. Within our polymorphic SSR set, (AG)_n_, (AAG)_n_, and (AAAG)_n_ are most prevalent in each repeat class (Table S3), a bias that was first recognized in *Arabidopsis* [85]. Within our full set of detected SSRs, motif type (AG/CT)_n_ comprises 70.4% of all di-nucleotide repeats, with the (CG/GC)_n_ motif nearly nonexistent (Fig. 4). The bias towards AG and against CG repeats has been demonstrated across eukaryotes [86], including within other legumes such as *Phaseolus* [87] and winged bean [29]. Prior studies have suggested that AG repeat motifs are commonly found in 5’ untranslated regions [84] and, as such, may be involved in transcription and regulation [85]. In the full set of detected SSRs, the (AAG/GTT)_n_ repeat motifs and their complements are the most prevalent. The ranking of tri-nucleotide repeat classes closely mirrors that found in winged bean [29].

Validation of SSRs discovered via transcriptome sequencing is the next step to building a working marker set for genetic improvement efforts. Of the 134 primer pairs we screened, over 73% successfully amplified genomic DNA across 25 *Mucuna* accessions. Our success rate is comparable to or somewhat lower than other efforts to validate transcriptome-derived SSR markers in legumes: Dutta *et al* [88] had a 80% success rate within pigeon pea; Liu *et al* [89] found 82% success in Alfalfa, whereas Jhanwar *et al* [90] found a high (98%) success rate in the cultivated chickpea. For genic-derived SSRs, marker dropout could be cause by chimeric primers, by the creation of primers across intron/exon splice sites, or by creation across alternative splice sites or chimeric transcripts.

### Assessing genetic diversity and population structure within Indian *Mucuna pruriens* accessions

The relative influence of different barriers to gene flow, such physical or geographical separation or incipient genetic or morphological changes that impact the ability to crossbreed, against promoters of gene flow, such as migration or the movement or interbreeding of individuals by human mediation, ultimately impacts genetic diversity within a species, particularly cultivated species [91]. Given the economic, medicinal and ethnobotanical importance of *M. pruriens*, assessing the genetic diversity within *M. pruriens* is an important endeavor. Our efforts to create and validate a database of potential SSR markers ultimately yielded 52 polymorphic markers for genetic diversity assessment within 23 Indian *M. pruriens* accessions representing all three *M. pruriens* varieties and sourced across India. All but two of the 52 markers showed adequate to high ability to discern ancestry based on the Shannon Information Content (I) [43] using the suggested cutoff of I ≥ 0.3 [92], attesting to the utility of these markers for genetic diversity assessment. The average I across all our markers is 0.78, a value much higher than the ancestral discerning power of RAPD markers in *M. pruriens* (average of 0.62 across 15 primer pairs) [93]. The average polymorphic information content (PIC) across our 52 markers in *M. pruriens* is 0.24, a value similar to that obtained by Leelambika et al. [94] but higher than all estimates using AFLP or RAPD data which ranged from 0.166 [95] to 0.174 [94], attesting to the appropriate choice of SSR markers for assessing genetic diversity in *Mucuna*.

We explored genetic diversity across different subpopulation divisions based on geography (East, Northeast, and Peninsular India), variety (*M.* vars. *pruriens, hirsuta,* and *utilis*), and empirical genetic structure (fastSTRUCTURE subgroups 1-4) (Table 6). East India showed a slightly higher gene diversity than other geographical areas, a finding somewhat surprising given that all accessions for East India are of a single variety, *M. p.* var. *pruriens*. Peninsular India had the highest average number of alleles, which could be attributed to this area comprising accessions from all three varieties, leading to an overall higher average number of alleles through inclusion of the allelic diversity specific to variety. Many cultivated crops are genetically depauperate compared to their wild relatives [96], and *M. p.* var. *utilis* is no exception. Wild accessions (*M.* vars. *pruriens* and *hirsuta*) had higher gene diversity and a higher average number of alleles than cultivated (*M. p.* var. *utilis*) accessions (Table 6), corroborating previous studies in *Mucuna* [93, 94]. Similar trends are found throughout legumes, for example within *Phaseolus vulgaris* [97]. However, estimates of gene flow were high regardless of subdivision type (i.e. geography, variety, genetic structure), suggesting significant mixing among germplasm. All that said, caution is warranted in interpreting these comparative results given our low number of accessions examined.

Estimation of population genetic substructure revealed *K* = 4 subgroups (Fig. 5a) with coefficient of ancestry placing most individuals strongly within a particular subgroup. Both geography and variety are somewhat correlated to groupings within genetic substructure, PCA, and clustering analyses, indicating their impact on structure of genetic diversity. For instance, subgroups 2, 3 and 4 contain genotypes mostly collected from east India, peninsular India + Maharashtra and northeast India, respectively, except for a few accessions whose placement was variable. Subgroup 2 comprises only var. *pruriens* accessions that are mostly from eastern India whereas subgroup 3 is mainly var. *hirsuta* with one accession each of var. *pruriens* and var. *utilis*.

Neither geography nor variety correlated completely with clades produced by the NJ algorithm, in contrast to previous cluster analyses based on ISSR data which were largely associated by taxonomy [94]. That said, a recent analysis using ISSR and RAPD markers across several species of *Mucuna,* including all *pruriens* varieties, found evidence via UPGMA cluster analyses for varietal cohesion of var. *utilis* and var. *pruriens*, as well as their sister relationship, but found that var. *hirsuta* clustered apart from the others, suggesting a separate evolutionary trajectory for this variety [98]. The lack of strict varietal clustering across our three population structure assessment methods (Fig. 5) coupled with high estimates of migration (Table 6) and clear suggestion of at least two hybrid individuals as determined by mixed coancestry within fastSTRUCTURE analyses suggests that hybridization can take place easily among varieties, a conclusion also suggested previously [94]. Alternatively, these variations may represent ancestral states perpetuated into extant populations. However, the small sample size used in this study resulted in limited power to accurately identify subgroups containing consistent genotypes across all the three methods tested. Thus, further studies involving larger samples derived from extended geographical regions are needed to make generalized conclusions on the divergence and population structure of Indian *M. pruriens*.

## Additional files

Additional file 1: Figures and tables highlighting the analysis and results obtained in this study. **Figure S1.** Length distribution of *Mucuna pruriens* transcripts in Trinity assembly. **Figure S2.** Functional characterization and abundance of *Mucuna pruriens* transcriptome for enzyme classes (A) and KEGG pathways (B). Transcripts were classified in the top 20 abundant enzyme classes and KEGG pathways; area under each pie slice represents the value in percent. **Figure S3.** Legume sequence similarity analysis. Percentage identity of transcripts against other legume protein databases (A) and relative numbers of transcripts that had significant sequence similarity by species (B). The percentage of transcripts showing similarity value (E-value ≤ 1E-05) in BLASTX searches is shown. **Figure S4.** Repeat distribution in *Mucuna pruriens* transcriptome discovered using lobSTR program. **Figure S5.** Results of fastSTRUCTURE analysis across K = 2 to K = 10 subgrounds. Subgroup number K plotted against marginal likelihood (A) and cross validation error (B). **Figure S6.** Heat map and complete linkage hierarchical clustering of differentially expressed transcripts of leaf, pod, root, and pooled transcriptomes. The various shades in the boxes showed similar tendencies of gene expression. **Table S1.** Contrasting phenotypes of the two genotypes used for transcriptome sequencing. **Table S2.** Length distribution of the EST-SSRs based on the number of nucleotide repeats. **Table S3.** Summary of the repeats in *Mucuna pruriens* transcriptome based on lobSTR. **Table S4.** Polymorphism information of 52 EST-SSR markers for 23 *Mucuna pruriens* accessions. Ho: Observed heterozygosity; He: Expected heterozygosity (Kimura & Crow, 1964); PIC: Polymorphism information content; Na: No. of alleles; Ne: Effective no. of alleles; I: Shannon information content; h: Nei’s (1973) gene diversity. (DOCX 4530 kb)

Additional file 2: Number of transcripts assigned to Biological, Cellular and Metabolic processes from GO analysis of the annotated transcripts of the *Mucuna* assembly. (XLSX 249 kb)

Additional file 3: Number of transcripts assigned for enzyme classes and KEGG pathways of the M.pruriens transcripts. (XLSX 26 kb)

Additional file 4: BLASTP analysis of the transcripts against the legume protein databases. (XLSX 4558 kb)

Additional file 5: Transcription factors identified in the *Mucuna* assembly using PlnTFDB (http://plntfdb.bio.uni-potsdam.de/v3.0, Pérez-Rodríguez et al. 2009) (XLSX 81 kb)

Additional file 6: Transcripts with the repeats in the Mucuna assembly identified using Perl script MISA (MicroSAtellite; http://pgrc.ipk-gatersleben.de/misa; Thiel et al. 2003) (XLSX 2581 kb)

Additional file 7: Description of the repeats identified in the assembly using MISA and the percentage of each class are reported. In addition the position of the start/end of the 7,943 repeat sequences is also reported. (XLSX 302 kb)

Additional file 8: Polymorphic sequence repeats identified between the *Mucuna* genotypes using lobSTR program, including the full list and the filtered list. (XLSX 545 kb)

Additional file 9: Fold change expression values of the transcripts in each of the tissue analyzed related to the secondary metabolite pathways. (XLSX 227 kb)

## Abbreviations

AFLP: Amplified fragment length polymorphism
ATP: Adenosine triphosphate
bHLH: Basic helix-loop-helix
BLAST: Best local alignment search tool
bp: Base pair
C: Chromosome
CAMTA: Calmodulin-binding transcription activator
cDNA: Coding deoxyribonucleic acid
CTAB: Cetyltrimethylammonium bromide
DET: Differentially expressed transcript
DNA RNA: Ribonucleic acid
DNA: Deoxyribonucleic acid
dNTP: Deoxynucleotide triphosphate
EC: Enzyme class
EST-SSR: Expressed sequence tag-simple sequence repeats
FPKM: Fragments per kilobase per million
GB: Gigabase
GO: Gene ontology
Gst: Coefficient of gene differentiation
h: Gene diversity
He: Expected heterozygosity
Ho: Observed heterozygosity
Hs: Gene diversity within groups
Ht: Total gene diversity
I: Shannon’s information index
K: Number of subpopulations
KEGG: Kyoto encyclopedia of genes and genomes
MADS: MCM1, AGAMOUS, DEFICIENS, and SRF box genes
MAF: Minor allele frequency
Mbp: Megabase pair
mRNA: Messenger RNA
MYB: Myeloblastosis
NAC: NAM, ATAF, and CUC
Ne: Effective number of alleles
NGS: Next generation sequencing
NJ: Neighbor-joining
Nm: Gene flow index
PC: Principal component
PCA: Principal component analysis
PCR: Polymerase chain reaction
PHD: Plant homeodomain
PIC: Polymorphic information content
RAPD: Random amplified polymorphic
SSR: Simple sequence repeat
TF: Transcription factor
VCF: Variant call format
