# Supplementary material for: Transcriptomic resources for the medicinal legume Mucuna pruriens: de novo transcriptome assembly, annotation, identification and validation of EST-SSR markers
Source: BMC Genomics. 2017 May 25;18:409. doi: 10.1186/s12864-017-3780-9 (PMC5445377; doi:10.1186/s12864-017-3780-9)
Supplement: Supplementary file 1 — Figures and tables highlighting the analysis and results obtained in this study. Figure S1. Length distribution of Mucuna pruriens transcripts in Trinity assembly. Figure S2. Functional characterization and abundance of Mucuna pruriens transcriptome for enzyme classes (A) and KEGG pathways (B). Transcripts were classified in the top 20 abundant enzyme classes and KEGG pathways; area under each pie slice represents the value in percent. Figure S3. Legume sequence similarity analysis. Percentage identity of transcripts against other legume protein databases (A) and relative numbers of transcripts that had significant sequence similarity by species (B). The percentage of transcripts showing similarity value (E-value ≤ 1E-05) in BLASTX searches is shown. Figure S4. Repeat distribution in Mucuna pruriens transcriptome discovered using lobSTR program. Figure S5. Results of fastSTRUCTURE analysis across K = 2 to K = 10 subgrounds. Subgroup number K plotted against marginal likelihood (A) and cross validation error (B). Figure S6. Heat map and complete linkage hierarchical clustering of differentially expressed transcripts of leaf, pod, root, and pooled transcriptomes. The various shades in the boxes showed similar tendencies of gene expression. Table S1. Contrasting phenotypes of the two genotypes used for transcriptome sequencing. Table S2. Length distribution of the EST-SSRs based on the number of nucleotide repeats. Table S3. Summary of the repeats in Mucuna pruriens transcriptome based on lobSTR. Table S4. Polymorphism information of 52 EST-SSR markers for 23 Mucuna pruriens accessions. Ho: Observed heterozygosity; He: Expected heterozygosity (Kimura & Crow, 1964); PIC: Polymorphism information content; Na: No. of alleles; Ne: Effective no. of alleles; I: Shannon information content; h: Nei’s (1973) gene diversity. (DOCX 4530 kb) [file 12864_2017_3780_MOESM1_ESM.docx]

**Supplementary Figure S1. Length distribution of *Mucuna pruriens* transcripts in Trinity assembly**.


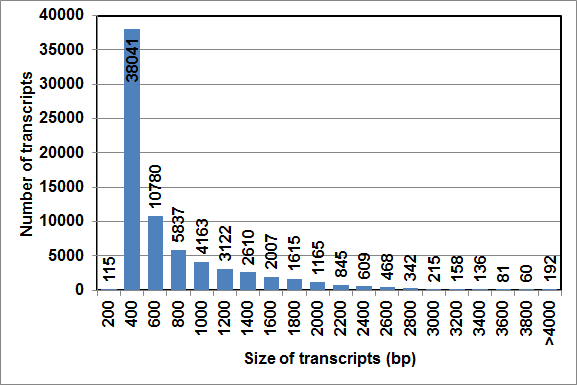


**Supplementary Figure S2. Functional characterization and abundance of *Mucuna pruriens* transcriptome for enzyme classes (A) and KEGG pathways (B).** Transcripts were classified in the top 20 abundant enzyme classes and KEGG pathways; area under each pie slice represents the value in percent.


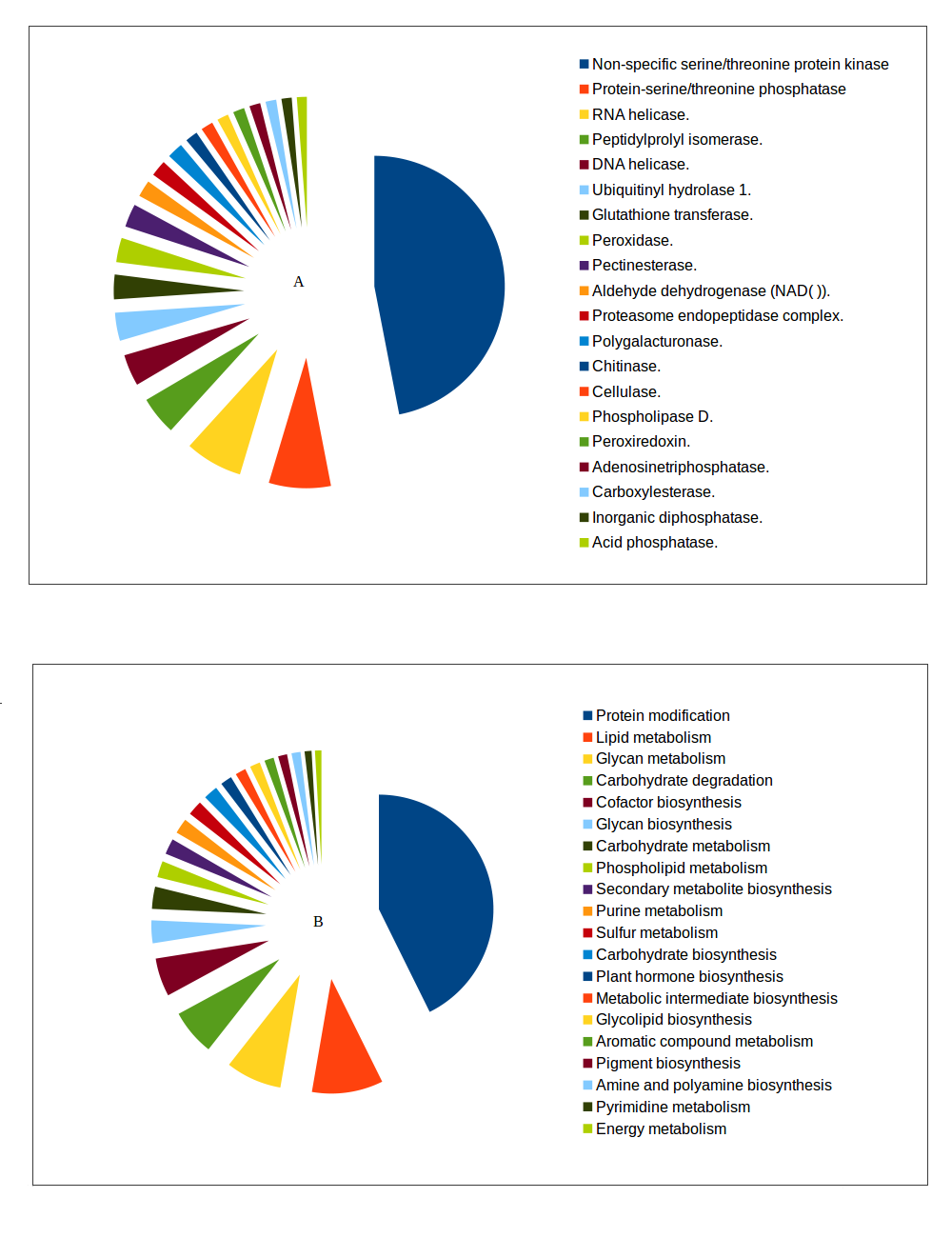


**Supplementary Figure S3. Legume sequence similarity analysis.** Percentage identity of transcripts against other legume protein databases (A) and relative numbers of transcripts that had significant sequence similarity by species (B). The percentage of transcripts showing similarity value (E-value ≤ 1E-05) in BLASTX searches is shown.

A.


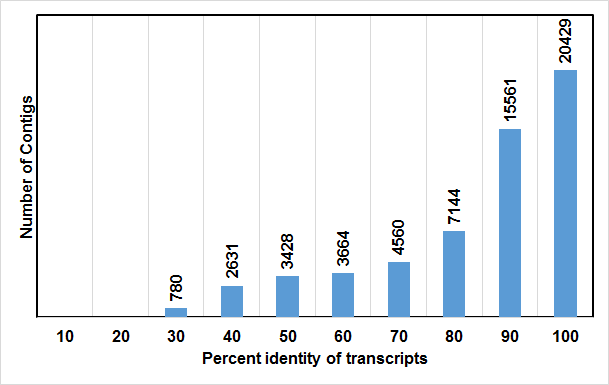


B.


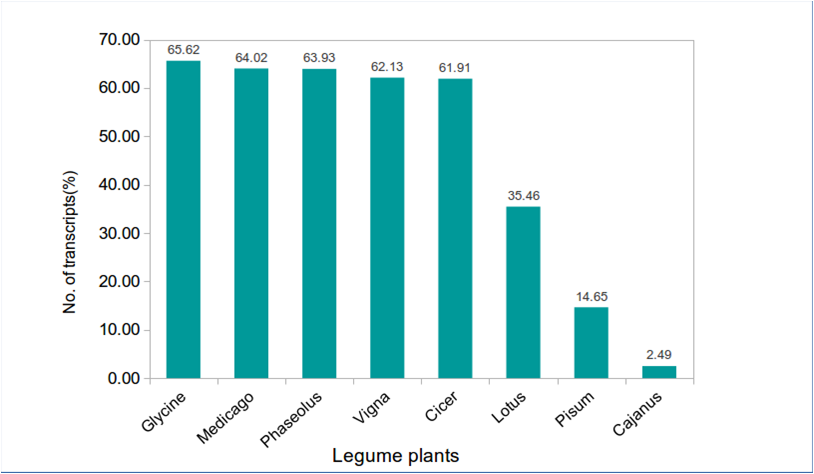


**Supplementary Figure S4. Repeat distribution in *Mucuna pruriens* transcriptome discovered using lobSTR program.**


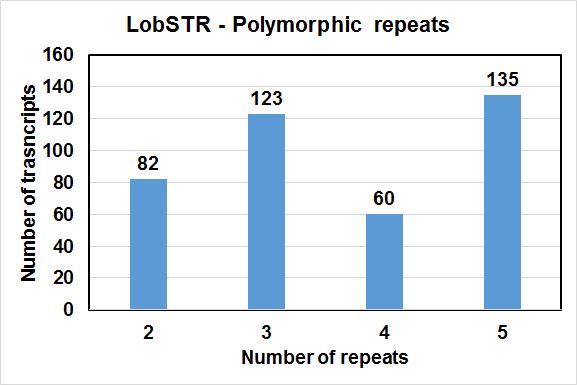


**Supplementary Figure S5. Results of fastSTRUCTURE analysis across K=2 to K=10 subgrounds.** Subgroup number K plotted against marginal likelihood (A) and cross validation error (B).


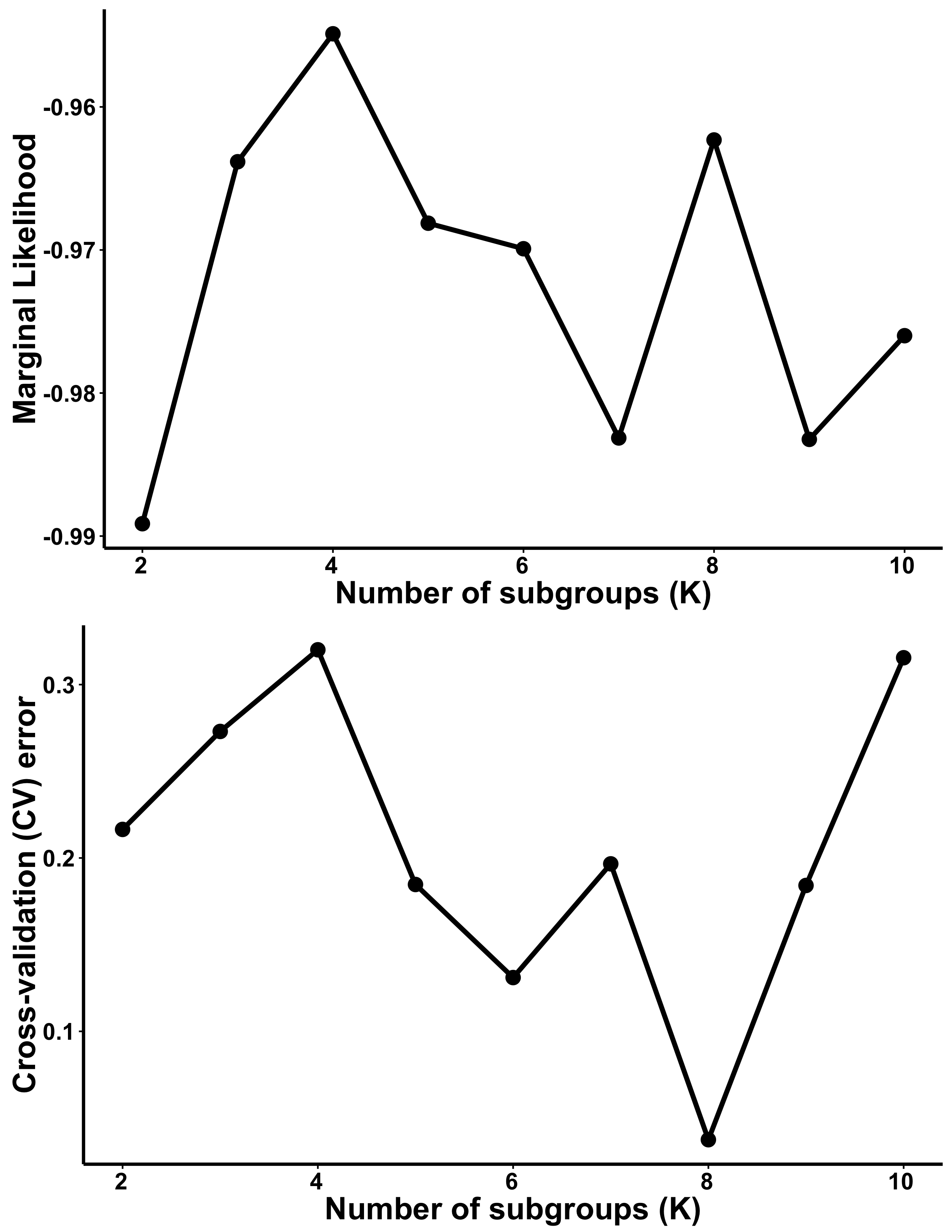


**Supplementary Figure S6. Heat map and complete linkage hierarchical clustering of differentially expressed transcripts of leaf, pod, and root transcriptomes**. The various shades in the boxes showed similar tendencies of gene expression.


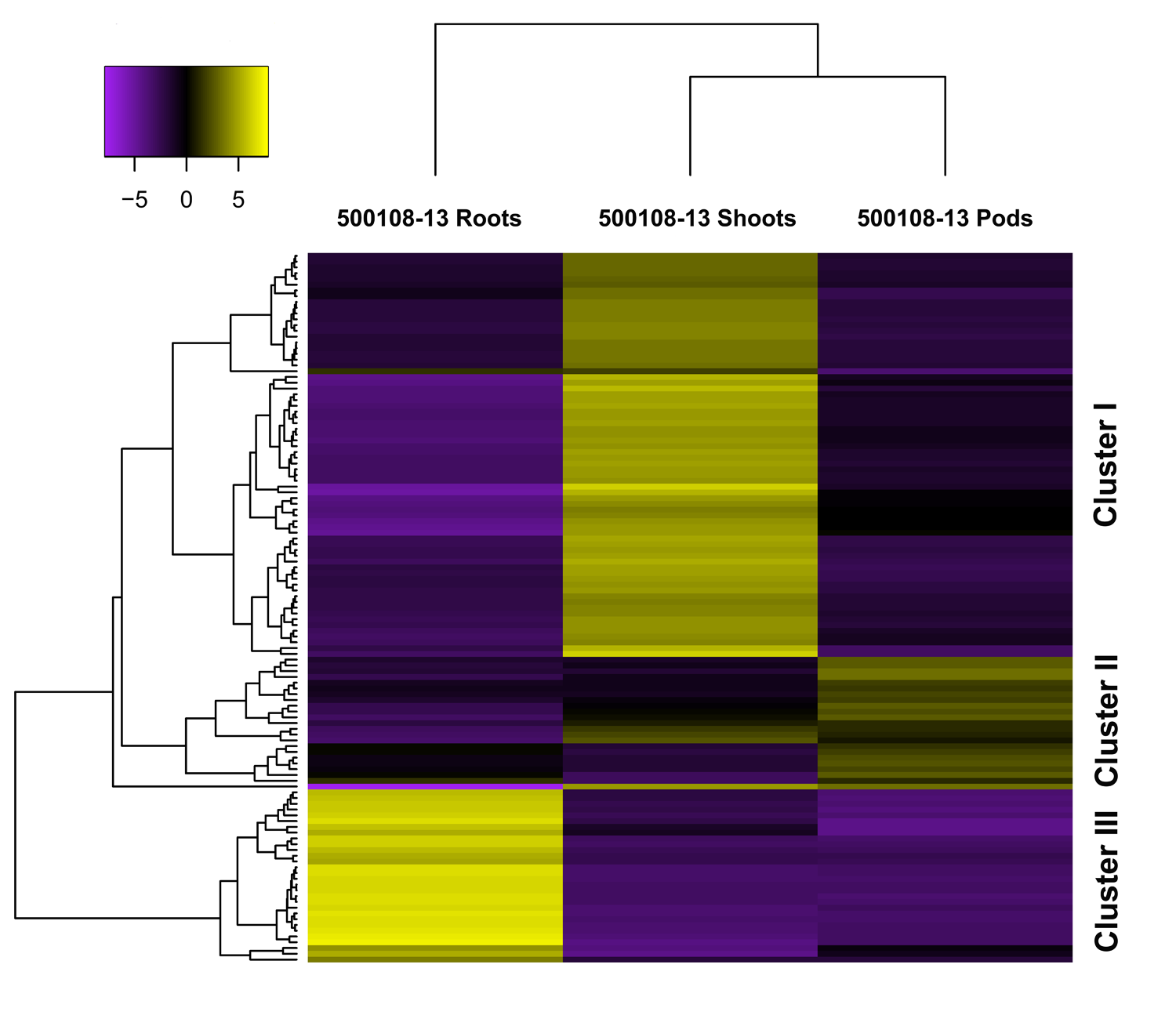


**Supplementary Table S1: Contrasting phenotypes of the two genotypes used for transcriptome sequencing.**

| Sl. No | Phenotype | Genotypes | |
| --- | --- | --- | --- |
|  |  | IC0620620  (G1; var. *utilis*♀) | IC0620622  (G2; var. *pruriens*♂) |
| 1 | Inflorescence length (cm) | 34.33 ± 4.99 | 93.00 ± 6.82 |
| 2 | No. of buds per cluster | 31.00 ± 3.74 | 86.75 ± 9.73 |
| 3 | Pod trichome color | Creamish white | Golden orange |
| 4 | Pod itchiness | Non-itching | Highly itching |
| 5 | No. of pods per plant | 203 | 340 |
| 6 | Days to maturity | 210 | 170 |
| 7 | Seed color | White | Brown (RHS 199C) |
| 8 | Weight of 100 seeds (g) | 93.05 ± 5.84 | 27.75 ± 2.598 |

G: genotype; No.: number.

**Supplementary Table S2: Length distribution of the EST-SSRs based on the number of nucleotide repeats.**

| No. of Repeats | Mono- | Di- | Tri- | Quad- | Penta- | Hexa- | Total** | Percentage (%) |
| --- | --- | --- | --- | --- | --- | --- | --- | --- |
| 5 |  |  | 1,269 | 96 | 47 | 59 | 1,471 | 18.71 |
| 6 |  | 611 | 537 | 36 | 11 | 20 | 1,215 | 15.45 |
| 7 |  | 329 | 240 | 7 | 3 | 12 | 591 | 7.51 |
| 8 |  | 260 | 124 | 2 | 2 | 2 | 390 | 4.9 |
| 9 |  | 163 | 20 | 2 | 0 | 0 | 185 | 2.35 |
| 10 | 1,238 | 108 | 18 | 2 | 0 | 5 | 1,371 | 17.43 |
| 11 | 653 | 65 | 13 | 0 | 0 | 1 | 732 | 9.31 |
| 12 | 393 | 58 | 9 | 0 | 0 | 0 | 460 | 5.85 |
| ≥13 | 1,354 | 80 | 10 | 1 | 1 | 1 | 1,447 | 18.40 |
| Total | 3,638 | 1,674 | 2,240 | 146 | 64 | 100 | 7,862 |  |
| Percentage | 45.80 | 21.07 | 28.02 | 1.83 | 0.80 | 1.25 |  |  |

**Data are given only up to hexa- nucleotide.

**Supplementary Table S3: Summary of the repeats in *Mucuna pruriens* transcriptome based on lobSTR.**

| **SSR Type** | **Repeat Motif** | **Repeat Size (bp)** | **Number of SSRs** | **% of total SSRs** |
| --- | --- | --- | --- | --- |
| **Di-nucleotide** | AG | 2 | 166 | 4.45 |
|  | AT | 2 | 27 | 0.72 |
|  | AC | 2 | 25 | 0.67 |
| **Tri-nucleotide** | AAG | 3 | 207 | 5.55 |
|  | ATC | 3 | 100 | 2.68 |
|  | AGG | 3 | 86 | 2.31 |
|  | AAC | 3 | 84 | 2.25 |
|  | AGC | 3 | 79 | 2.12 |
|  | ACC | 3 | 61 | 1.64 |
|  | CCG | 3 | 58 | 1.55 |
|  | AAT | 3 | 42 | 1.13 |
|  | ACG | 3 | 23 | 0.62 |
|  | ACT | 3 | 19 | 0.51 |
| **Tetra-nucleotide** | AAAG | 4 | 52 | 1.39 |
|  | AAAT | 4 | 29 | 0.78 |
|  | AAAC | 4 | 28 | 0.75 |
|  | AATG | 4 | 17 | 0.46 |
|  | ACTC | 4 | 17 | 0.46 |
|  | ACAT | 4 | 16 | 0.43 |
|  | AGGG | 4 | 12 | 0.32 |
|  | AAGG | 4 | 12 | 0.32 |
|  | AACC | 4 | 10 | 0.27 |
|  | AGAT | 4 | 9 | 0.24 |
|  | AATC | 4 | 9 | 0.24 |
|  | AATT | 4 | 8 | 0.21 |
|  | AAGC | 4 | 7 | 0.19 |
|  | ACCC | 4 | 5 | 0.13 |
|  | AGCT | 4 | 5 | 0.13 |
|  | ACGC | 4 | 4 | 0.11 |
|  | AACT | 4 | 4 | 0.11 |
|  | ATGC | 4 | 4 | 0.11 |
|  | ATCC | 4 | 3 | 0.08 |
|  | AGCG | 4 | 2 | 0.05 |
|  | AGGC | 4 | 2 | 0.05 |
|  | ATCG | 4 | 1 | 0.03 |
|  | ACGT | 4 | 1 | 0.03 |
|  | ACTG | 4 | 1 | 0.03 |
|  | ACCG | 4 | 1 | 0.03 |
|  | ACCT | 4 | 1 | 0.03 |
|  | CCCG | 4 | 1 | 0.03 |
| **Penta-nucleotide** | Total | 5 | 606 | 16.25 |
| **Hexa-nucleotide** | Total | 6 | 1886 | 50.56 |

**Supplementary Table S4: Polymorphism information of 52 EST-SSR markers for 23 *Mucuna pruriens* accessions.**

|  | **Primer ID** | **Forward Primer** | **Reverse Primer** | **Motif** | **alleles** | **Na** | **Ne** | **I** | **Ho** | **He** | **h** | **PIC** |
| --- | --- | --- | --- | --- | --- | --- | --- | --- | --- | --- | --- | --- |
| 1 | MPU_06 | GCATTTCAAATAAACGTCAAC | TTGACACAATTTGACAGAGGT | AGG | 3 | 2 | 1.32 | 0.41 | 0.1 | 0.25 | 0.23 | 0.19 |
| 2 | MPU_07 | AATGGATCCCTTTTCTCTATG | TATTGGAATAGATCCCCTTGT | ACA | 2 | 3 | 1.38 | 0.5 | 0.04 | 0.27 | 0.29 | 0.23 |
| 3 | MPU_15 | AGTTAAAACTCACCCACCCTA | TGGAGGTGAGTGAATAGATGA | TC | 3 | 5 | 2.5 | 1.16 | 0.05 | 0.6 | 0.26 | 0.27 |
| 4 | MPU_16 | GACTCCAACTCCTCCTTCA | ACTGTTGTTGTTGCTGATGTT | ACA | 4 | 4 | 2.32 | 1.1 | 0.24 | 0.57 | 0.2 | 0.24 |
| 5 | MPU_18 | GAAGAGGAGGTTCAGAACAGT | GCATCAGATACAACAAAGGAG | GAA | 2 | 2 | 1.54 | 0.54 | 0 | 0.35 | 0.35 | 0.35 |
| 6 | MPU_21 | CCGAGAGTACAAGGGTAAAAA | TTCTCCTTTCCCTGAAGATAC | GAA | 1 | 3 | 1.2 | 0.37 | 0 | 0.17 | 0.28 | 0.15 |
| 7 | MPU_22 | TAAAACTCCTTTTCCTTCTCC | AGTTCCTTCAAAATACGCTTC | TC | 3 | 4 | 3.04 | 1.17 | 0.09 | 0.67 | 0.28 | 0.33 |
| 8 | MPU_23 | AAGCTTGTCACTGTCAAAAAG | ATGCAACACATGTCAACACT | CCT | 2 | 3 | 1.31 | 0.47 | 0 | 0.23 | 0.25 | 0.17 |
| 9 | MPU_24 | CATATCCTAGACCGCAGTTG | CGTTTTGGTCATTGTGAAT | ATA | 2 | 3 | 2.1 | 0.85 | 0.04 | 0.52 | 0.38 | 0.35 |
| 10 | MPU_27 | ATGAGAGTCTGAGGGAGATTG | ATGTTGCATTGTCTCAACAGT | AGA | 1 | 5 | 1.37 | 0.6 | 0.09 | 0.27 | 0 | 0.15 |
| 11 | MPU_30 | AAAAACTTTGCAGTACCGATT | TGATTCATTCAAAATCTCCAC | AT | 2 | 4 | 1.77 | 0.8 | 0.04 | 0.44 | 0.35 | 0.3 |
| 12 | MPU_31 | TGTCATTGGCTTGATATTCTT | GGAACGGAAATAAATTCTTGT | CAT | 3 | 6 | 2.5 | 1.24 | 0.24 | 0.6 | 0.26 | 0.28 |
| 13 | MPU_33 | CCAGCTTACAACAACAGTCTT | AACTTTGGACAGGAATATTGG | GCT | 2 | 2 | 1.29 | 0.39 | 0 | 0.23 | 0.27 | 0.2 |
| 14 | MPU_37 | GTTCTCCTTCTCTGGTGAGTC | CTAAGACAAGCACACCTCAAG | GTT | 2 | 2 | 1.63 | 0.57 | 0 | 0.39 | 0.37 | 0.31 |
| 15 | MPU_39 | AGTGGCAACTATATGACGGTA | GAGTCACAACCAAATAGCTCA | CA | 2 | 5 | 1.92 | 0.96 | 0 | 0.48 | 0.37 | 0.29 |
| 16 | MPU_42 | CAAAGATGACGAAGATGGATA | TCTTTCAACTCAATCTTCCAA | AG | 6 | 6 | 5.56 | 1.75 | 0.09 | 0.82 | 0.15 | 0.22 |
| 17 | MPU_43 | GTCTAGGGTGGCTTTGTAGAC | CAACACCCACCAAATCTAGTA | ACT | 2 | 3 | 1.33 | 0.47 | 0.05 | 0.25 | 0.05 | 0.08 |
| 18 | MPU_45 | ATCTCTGTCTCTGTCCCTTTC | GCGATCTCTCTCTTTCTCTCT | GA | 3 | 3 | 2.14 | 0.91 | 0.04 | 0.53 | 0.28 | 0.28 |
| 19 | MPU_47 | GTTCACCACATCCATTATGAC | ACCACACACACACATACACAC | TG | 2 | 2 | 1.51 | 0.52 | 0.05 | 0.34 | 0.35 | 0.28 |
| 20 | MPU_48 | GGTGGAGACCTTACACAATATG | CACCTCCAGTTTATTCACCTC | GAG | 4 | 4 | 2.33 | 1.01 | 0.22 | 0.57 | 0.22 | 0.27 |
| 21 | MPU_49 | GGAGGATAAAAGTGCTTCATT | ACCGTCCTCTTAATTGTTGAT | TG | 2 | 5 | 2.85 | 1.23 | 0 | 0.65 | 0.36 | 0.36 |
| 22 | MPU_52 | AAGGAAAGCATATGAAAGGAG | ATTCCTCCAATTCTATCTCGT | GGT | 3 | 6 | 2.15 | 1.08 | 0.33 | 0.53 | 0.22 | 0.25 |
| 23 | MPU_57 | TATCGATCTCAATTCCCATAA | TCTCTCTCTAAAGCCCAAAAC | GCG | 2 | 5 | 1.6 | 0.8 | 0.17 | 0.37 | 0.29 | 0.23 |
| 24 | MPU_58 | CACTCTCAGTGAGTCTTCTGG | GAATGCTTGAGGCATAGAAG | CAA | 2 | 2 | 1.29 | 0.39 | 0 | 0.23 | 0.24 | 0.2 |
| 25 | MPU_59 | GAGGTGTTGAAGAAGTGTGAG | ATTCCCACCATTCTCATTAGT | AGA | 3 | 3 | 1.36 | 0.49 | 0.13 | 0.26 | 0.2 | 0.2 |
| 26 | MPU_64 | GCTTTCTATGAATGACTGCAT | AATTTCCAACCATGGATTAAG | AT | 3 | 3 | 2.47 | 1 | 0.05 | 0.6 | 0.28 | 0.31 |
| 27 | MPU_65 | CCAGTTTAGCTTGCACATACT | CCAATAGGCGAATTATATGAC | CAT | 4 | 4 | 2.93 | 1.19 | 0.4 | 0.66 | 0.2 | 0.26 |
| 28 | MPU_68 | GGGTTAGGGTTACGGAAA | GCGATACTTCCTTTTCAAGTC | AG | 3 | 4 | 1.72 | 0.74 | 0.18 | 0.42 | 0.26 | 0.27 |
| 29 | MPU_72 | AAGAGCGATAGAGGAAGACAT | TGAAGTAGATTGGCAGGTTAG | TAG | 2 | 2 | 1.57 | 0.55 | 0.04 | 0.36 | 0.33 | 0.3 |
| 30 | MPU_78 | TGAAGAGCTTTCAAAGTGAAG | GAACGAATTTTTAGGGTGATT | CAA | 2 | 4 | 2.24 | 0.94 | 0.1 | 0.55 | 0.37 | 0.36 |
| 31 | MPU_80 | ACCAGAAAGGTAAAGCTCTCA | TTTTTGTTACTCTCCAAGCAG | GA | 1 | 4 | 1.22 | 0.41 | 0.09 | 0.18 | 0.48 | 0.21 |
| 32 | MPU_81 | TTCTATCGGAGAAGAAGTCG | CACACCTCTCTCTCTCTCTCTC | GA | 2 | 3 | 1.36 | 0.52 | 0 | 0.26 | 0.27 | 0.19 |
| 33 | MPU_83 | CACTCATGGAAGGTTCTAGGT | CTCATTTTGCTTTTCAAGAGA | CTT | 1 | 4 | 1.2 | 0.4 | 0.09 | 0.17 | 0 | 0.21 |
| 34 | MPU_88 | GGAACTGGGTATGAGGTATTT | AGATGAGACCCCATTTGTTAC | TC | 2 | 4 | 1.52 | 0.65 | 0.09 | 0.34 | 0.31 | 0.25 |
| 35 | MPU_89 | ATAGCTCCGATTACCCAAAT | CACCCATTTCTCTCTTTCTCT | TGGGTT | 2 | 3 | 2.06 | 0.78 | 0.09 | 0.52 | 0.23 | 0.21 |
| 36 | MPU_90 | TTCCAAACAGTGGTCATAATC | TGTATTGCTGTTGGTAAGGAT | GGT | 4 | 5 | 4.03 | 1.45 | 0.46 | 0.75 | 0.16 | 0.26 |
| 37 | MPU_92 | CGGTCGTAGTAATCGCTTC | GGTTTTGCTCTGTTTTCTCTT | GGA | 3 | 4 | 2.4 | 1.04 | 0.35 | 0.58 | 0.24 | 0.3 |
| 38 | MPU_98 | TGCCTTATGAGGAATCTTACA | GTATTTCCCCTTGTTCATCTT | AAC | 2 | 3 | 1.56 | 0.66 | 0.05 | 0.36 | 0.31 | 0.26 |
| 39 | MPU_99 | ACTGCAGAATGGAACTTTGTA | TAAAAGCCCTACTCAAAATCC | TC | 3 | 4 | 1.61 | 0.75 | 0.09 | 0.38 | 0.24 | 0.26 |
| 40 | MPU_102 | GAGAGAACTTCAATTCCCCTA | GCTTGTTCATGTTTCTTTGTC | CT | 1 | 6 | 1.44 | 0.7 | 0.13 | 0.31 | 0.5 | 0.28 |
| 41 | MPU_106 | GGAGAAATCAAATAGGTCGTC | AGTTCAAACCTAACCGAACTC | AGA | 2 | 3 | 1.4 | 0.53 | 0.04 | 0.28 | 0.27 | 0.2 |
| 42 | MPU_107 | CGTATTTGTTGAACAGGATGT | GTCTCTCCTACCTCGATCAGT | CTGC | 3 | 6 | 4.1 | 1.54 | 0.25 | 0.76 | 0.19 | 0.26 |
| 43 | MPU_111 | CATCAACCTTATTCGACTCAT | AGTTCCTCCGTTACGTCAT | GCC | 5 | 5 | 3.1 | 1.32 | 0.32 | 0.68 | 0.17 | 0.24 |
| 44 | MPU_114 | GTCTCTTTCACTCTTCCGTTT | GCAAGGTCTCTTTCACTCTTT | TTC | 2 | 2 | 1.31 | 0.4 | 0 | 0.24 | 0.24 | 0.21 |
| 45 | MPU_115 | AAAGGCAATTGAGATCTAAGAA | CCTTTGTCTCCTCTTTCTTTC | AG | 3 | 4 | 2 | 0.95 | 0.05 | 0.5 | 0.23 | 0.22 |
| 46 | MPU_119 | GGGTTTGACAGGAAGTACAA | ACTTCCTAGAGCTTCCACTGT | AGA | 1 | 2 | 1.1 | 0.2 | 0 | 0.1 | 0 | 0.09 |
| 47 | MPU_121 | TCATCACCATCACCATCAC | GATATCTGCCAGGTCCATC | CAC | 2 | 4 | 1.44 | 0.64 | 0 | 0.31 | 0.27 | 0.2 |
| 48 | MPU_122 | TTCCTCGTGGAGATACAGATA | TGATGCATGCTATGATTAGAA | CAG | 1 | 3 | 1.14 | 0.28 | 0.04 | 0.12 | 0 | 0.15 |
| 49 | MPU_123 | TTGTGCTGTTGTTATGATTGA | CTTTGTTTAAAACTGAACCA | AAT | 1 | 4 | 1.37 | 0.57 | 0.04 | 0.27 | 0.5 | 0.25 |
| 50 | MPU_124 | CCTTCCCTTTAGATGTGAAAT | ACATTGATAGCAGTGGAGAAA | GCA | 4 | 6 | 5.01 | 1.7 | 0.04 | 0.8 | 0.2 | 0.27 |
| 51 | MPU_125 | AGGAGAGAGAGTGAAATTGGA | ACAACGTGAACAGAGAGAGAA | AGA | 1 | 4 | 1.31 | 0.53 | 0 | 0.24 | 0.42 | 0.2 |
| 52 | MPU_126 | TCTATGGGTTTGTCCTCATC | CATTTTTCCACAATCACTTTC | GTG | 2 | 2 | 1.29 | 0.39 | 0.17 | 0.23 | 0.28 | 0.26 |
|  | **Average** | - | - | - | **2.4** | **3.73** | **1.98** | **0.78** | **0.1** | **0.41** | **0.26** | **0.24** |
|  | **SD** | - | - | - | **1.07** | **1.25** | **0.96** | **0.37** | **0.11** | **0.19** | **0.11** | **0.06** |

Ho- Observed heterozygosity; He- Expected heterozygosity (Kimura & Crow, 1964); PIC-Polymorphism information content; Na- No. of alleles; Ne- Effective no. of alleles; I- Shannon information content; h- Nei’s (1973) gene diversity.
